# Transcription factors LvBBX24 and LvbZIP44 coordinated anthocyanin accumulation in response to light in lily petals

**DOI:** 10.1093/hr/uhae211

**Published:** 2024-07-30

**Authors:** Zhenhua Gao, Yibo Sun, Ziman Zhu, Na Ni, Shaokun Sun, Mengyao Nie, Weifeng Du, Muhammad Irfan, Lijing Chen, Li Zhang

**Affiliations:** Key Laboratory of Agriculture Biotechnology, Key Laboratory of Protected Horticulture (Ministry of Education), College of Biosciences and Biotechnology, Shenyang Agricultural University, Shenyang, Liaoning 110161, China; Key Laboratory of Agriculture Biotechnology, Key Laboratory of Protected Horticulture (Ministry of Education), College of Biosciences and Biotechnology, Shenyang Agricultural University, Shenyang, Liaoning 110161, China; Key Laboratory of Agriculture Biotechnology, Key Laboratory of Protected Horticulture (Ministry of Education), College of Biosciences and Biotechnology, Shenyang Agricultural University, Shenyang, Liaoning 110161, China; Key Laboratory of Agriculture Biotechnology, Key Laboratory of Protected Horticulture (Ministry of Education), College of Biosciences and Biotechnology, Shenyang Agricultural University, Shenyang, Liaoning 110161, China; Institute of Vegetable Research, Liaoning Academy of Agricultural Sciences, Shenyang, Liaoning 110161, China; Key Laboratory of Agriculture Biotechnology, Key Laboratory of Protected Horticulture (Ministry of Education), College of Biosciences and Biotechnology, Shenyang Agricultural University, Shenyang, Liaoning 110161, China; Key Laboratory of Agriculture Biotechnology, Key Laboratory of Protected Horticulture (Ministry of Education), College of Biosciences and Biotechnology, Shenyang Agricultural University, Shenyang, Liaoning 110161, China; Department of Biotechnology, University of Sargodha, Sargodha Pakistan; Key Laboratory of Agriculture Biotechnology, Key Laboratory of Protected Horticulture (Ministry of Education), College of Biosciences and Biotechnology, Shenyang Agricultural University, Shenyang, Liaoning 110161, China; Key Laboratory of Agriculture Biotechnology, Key Laboratory of Protected Horticulture (Ministry of Education), College of Biosciences and Biotechnology, Shenyang Agricultural University, Shenyang, Liaoning 110161, China

## Abstract

Lily (*Lilium* spp*.*), a horticultural crop serving both ornamental and edible functions, derives its coloration primarily from anthocyanins. However, limited studies have been conducted on the accumulation of anthocyanins within lilies. In this study, we cloned a light-induced transcription factor named as LvBBX24 in lilies. Through genetic and biochemical analysis, we determined that LvBBX24 could upregulate the transcription of *LvMYB5* and facilitate anthocyanin synthesis. Moreover, we identified that darkness promoted the degradation of LvBBX24 protein. Through screening a yeast library, we identified LvbZIP44 acts as its interacting partner. Genetic testing confirmed that LvbZIP44 also plays a role in promoting lily anthocyanin synthesis. This indicates a potential synergistic regulatory effect between LvBBX24 and LvbZIP44. Our study indicates that LvBBX24 and LvbZIP44 cooperate to regulate anthocyanin accumulation in lily petals. These findings provide compelling evidence supporting the idea that LvBBX24 and LvbZIP44 may form a looped helix surrounding the *LvMYB5* promoter region to regulate anthocyanin biosynthesis.

## Introduction

Lily (*Lilium* spp.) belongs to the Liliaceae family and is one of the five major cut flowers used globally. This ornamental plant serves three primary functions: ornamental, edible, and medicinal [1]. Anthocyanins, the primary color-producing substances in lilies, have a wide range of applications. These compounds aid in resistance to various stresses [2]. In addition, the consumption of anthocyanin-rich plants by humans can mitigate the adverse effects of different diseases [3, 4]. As a prevalent ornamental plant, the color of lilies is an important characteristic. The pigments predominantly consist of flavonoids and carotenoids, with anthocyanin being the primary color substance responsible for pink, purple, and other flower colorations [5]. Anthocyanins serve as secondary metabolites with antioxidant functions and play a crucial role in plant defense against environmental stresses [6].

The anthocyanin synthesis pathway is a critical component of the flavonoid synthesis pathway that is highly conserved in higher plants. This pathway involves multiple complex enzymatic reactions and can be separated into three primary stages: formation of the basic anthocyanin skeleton, production of cyanin precursors within flowers, and the modification of anthocyanin precursors into diverse classes of anthocyanins. The anthocyanin synthesis genes are classified into early biosynthetic genes (EBGs) and late biosynthetic genes (LBGs). The EBGs include chalcone synthase (*CHS*), chalcone isomerase (*CHI*), flavanone 3-hydroxylase (*F3H*), and flavonoid 3′-hydroxylase (*F3’H*), while the LBGs include dihydroflavonol-4-reductase (*DFR*), anthocyanin synthetase (*ANS*), and flavonoid-3-O-glucosyltransferas (*UFGT*) [7]. The synthesis of anthocyanin necessitates transporters and transport vesicles acting for effective transportation and storage into vacuoles. This process limits the oxidative denaturation of anthocyanins and their potential toxicity to cells [8–10].

Transcription factors are essential for regulating the functions of anthocyanins. The genes involved in the anthocyanin biosynthetic pathway are typically regulated by multiple transcription factors [11–13]. The most widespread transcription factors in this context are MYB, bHLH, and WDR [14]. These three classes of transcription factors can generate the MBW complex. R2R3-MYB regulatory factors, including Yamagishi [15], play a significant role in this process. For example, LvMYB5 in lily positively regulates the accumulation of anthocyanins [16]. In roses, light can impact the balance between RhMYB114 and RhMYB3b in light-mediated anthocyanin biosynthesis [17]. Moreover, other factors such as miRNA [18], DNA methylation [19], and histone modification [20] are involved in regulating anthocyanin synthesis.

**Figure 1:**
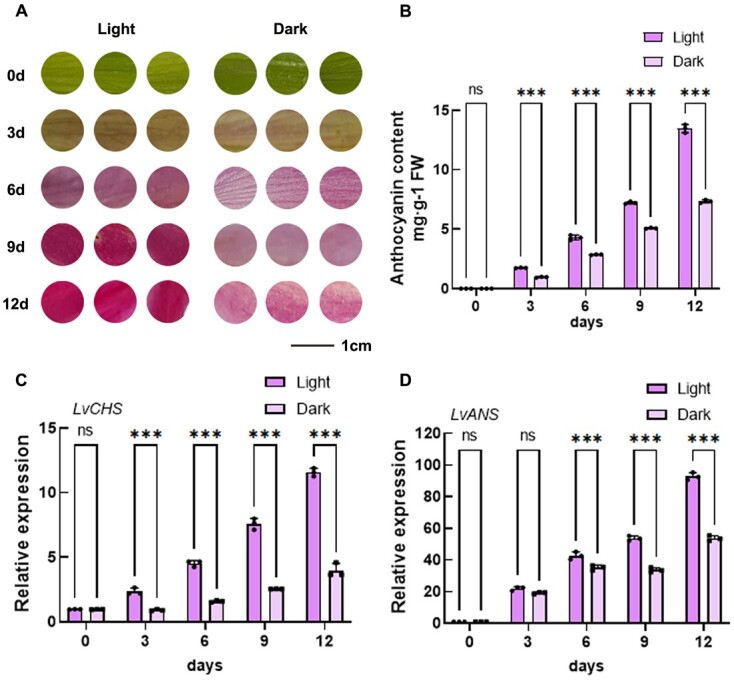
Induction of anthocyanin accumulation in lily petals through light treatment. **A** Phenotype of lily cultivar ‘Viviana’ petals after exposure to light treatment. Scale bar = 1 cm. **B** Quantification of anthocyanin content in light-treated lily petals. **C** and **D** Transcription levels of anthocyanin biosynthetic genes *LvCHS* and *LvANS* in light-treated lily petals. Data are the means of three biological replicates ± SD. Asterisks indicate significant differences (Student’s *t* test, ^***^P < 0.001; n.s., no significant difference).

The environment represents a crucial and influential factor for anthocyanin synthesis [21–23]. Among its influences, light plays the most significant role [24]. Several studies have underscored the interaction between MYB75 and SAP and Miz1 (SIZ1) in *Arabidopsis*, contributing to light-induced anthocyanin accumulation [25]. Additionally, in eggplant, SmMYB5 and SmJAZ5/10 interact under light conditions, incorporating jasmonic acid and anthocyanin biosynthesis when responding to light signals [26].

B-BOX proteins (BBXs) are broadly present in animals and plants, and their structure is stabilized by the binding of zinc ions [27]. In *Arabidopsis*, the first B-box-containing protein identified was CO/BBX1, which helps to induce flowering under long-day conditions [28]. Apart from their involvement in flowering, BBXs proteins also affect plant photomorphogenesis. Specifically, B-box proteins BBX21 and BBX23 positively regulate photomorphogenesis in response to light signals [29, 30].

Recently, a growing recognition of the impact of BBX on plant anthocyanin accumulation has been developed [31]. The apple B-box protein MdBBX37 plays a role in regulating jasmonic acid-mediated cold tolerance via the JAZ-BBX37-ICE1-CBF pathway. Additionally, it undergoes MIEL1-mediated ubiquitination and degradation [32]. BBX is a vast family of proteins in plants. While it is likely involved in anthocyanin synthesis, the specific mechanism, particularly in lilies, remains unclear. Therefore, further investigation is required to explore the role of BBX in monocot plants.

In this study, we isolated a light-responsive BBXs protein named as LvBBX24 from lily, which not only forms a heterodimer with LvbZIP44 to activate the transcription of the anthocyanin activator *LvMYB5* but also acts independently on the promoter of *LvMYB5*, enhancing anthocyanin accumulation. In addition, our results emphasize the significance of LvbZIP44 in promoting anthocyanin accumulation by binding to the *LvMYB5* promoter. This study shows potential physical interactions between BBX and bZIP transcription factors that increase anthocyanin synthesis in the light signaling pathway via a novel molecular mechanism.

## RESULTS

### Light treatment encourages the accumulation of anthocyanins in lily petals

Several studies have demonstrated that light can influence the levels of anthocyanins [26]. In our research, we identified similar outcomes in lilies. The presence of anthocyanins was notably suppressed in darkness (Fig. 1A), which was mirrored by the content of anthocyanins (Fig. 1B). Comparatively, lily petals exposed to light appeared significantly darker than those maintained in darkness, with their anthocyanin content displaying a significant disparity. Furthermore, the expression of anthocyanin synthesis genes (*LvCHS*, *LvANS*) exhibited a substantial elevation under light exposure, and *CHS* (Fig. 1C) experienced a threefold increase and *ANS* exhibited a twofold increase in expression levels (Fig. 1D).

### Light impacts the expression of *LvBBX24* and regulates the accumulation of anthocyanins in lily petals

**Figure 2:**
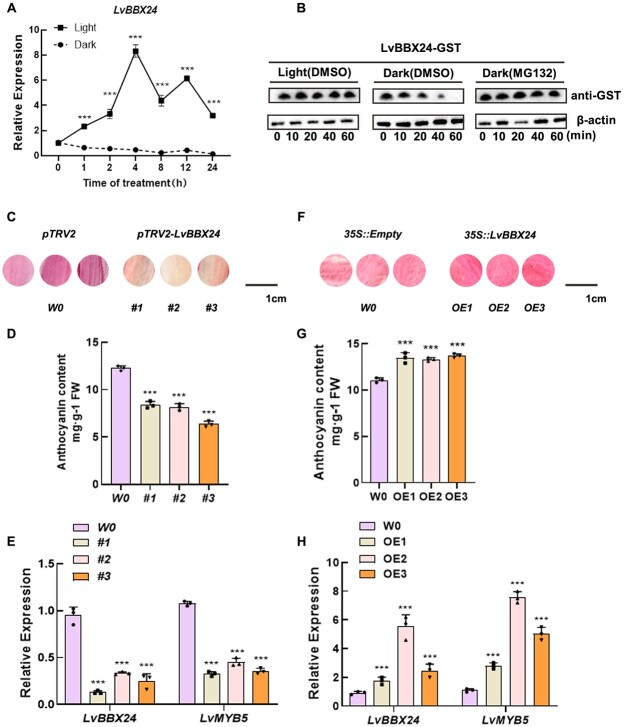
The impact of LvBBX24 on anthocyanin accumulation in lily petals under different light conditions was investigated. **A**  *LvBBX24* expression pattern was assessed after light treatment. **B**  *In vitro* degradation experiments were conducted to explore variations in LvBBX24-GST protein levels in response to light and dark conditions. Total protein was isolated and combined with either DMSO or MG132. Samples were harvested at specific time points (0, 10, 20, 40, and 60 minutes), utilizing actin as the internal reference. **C** Anthocyanin accumulation in lily petals was measured after transient silencing of *LvBBX24*. **D** and **E** The anthocyanin content in lily petals was determined after transient silencing of *LvBBX24*, along with the relative expression of *LvBBX24* and *LvMYB5*. **F** Anthocyanin accumulation in lily petals was assessed after transient overexpression of *LvBBX24*. **G** and **H** The anthocyanin content of lily petals was measured after transient overexpression of *LvBBX24*, along with the relative expression of *LvBBX24* and *LvMYB5*. *LvACTIN* was used as the internal control. Data in (A–I) are means ± SD (*n* = 3). Asterisks in A and D–H indicate significant differences (Student’s *t* test, ^***^P < 0.001).

To further examine light-induced anthocyanin accumulation, we performed transcriptome sequencing (RNA-seq) on petals exposed to light at different time points (NCBI project: PRJNA1113360). Through our analysis, we determined that the expression levels of *BBXs* are regulated by light (Fig. S1F, see online supplementary material). RT-qPCR findings revealed that the *BBX* gene responds differently to light (Fig. S1A–E, see online supplementary material). The BBXs were temporarily silenced in our study, revealing that the gene *TRINITY_DN97273_c0_g3* plays a role in the biosynthesis of anthocyanins in lily petals. Silencing this gene led to a notable decrease in anthocyanin accumulation, as well as a significant reduction in the expression levels of anthocyanin-related structural genes. Furthermore, a phylogenetic analysis of this BBX gene was conducted (Fig. S2, see online supplementary material). Phylogenetic analysis shows a close genetic relationship with AtBBX24 and AtBBX25, and sequence alignment is more similar to AtBBX24. We therefore named it LvBBX24 (Fig. S3A, see online supplementary material). Remarkably, as the illumination duration increased, the expression level of *LvBBX24* significantly increased (Fig. 2A). Further investigation suggested that LvBBX24 is localized in the nucleus (Fig. S3B, see online supplementary material). Furthermore, our analysis uncovered that LvBBX24-BD possesses potential transcription activation capacity, as it activates the -Trp (x -α-gal) reporter gene, generating a blue color (Fig. S3C, see online supplementary material).

In our study, we analysed the protein abundance of LvBBX24 under varying lighting conditions. Notably, we observed that LvBBX24 had a higher degradation rate when subjected to darkness (Fig. 2B). The results show that light has two effects on LvBBX24: it can induce the transcription of *LvBBX24* and promote its expression, and it can also inhibit the degradation of the protein. We speculate that the level of LvBBX24 protein may be related to the accumulation of anthocyanins. Building on these findings, we hypothesized that LvBBX24 plays a role in the biosynthesis of anthocyanins upon exposure to light stimulation. To examine this hypothesis, we conducted transient gene silencing experiments in lily petals (Fig. 2C). Remarkably, the silencing of *LvBBX24* caused a significant reduction in anthocyanin content (Fig. 2D). Furthermore, we performed RT-qPCR analysis and identified a substantial reduction in the expression level of *LvMYB5* upon silencing of *LvBBX24* (Fig. 2E). Conversely, when *LvBBX24* was transiently overexpressed, we identified a significant increase in anthocyanin content in the 35S::*LvBBX24* treatment (Fig. 2F–H).

### LvBBX24 positively regulates anthocyanin accumulation in lily petals by binding to the *LvMYB5* promoter

Previously, we identified a R2R3-MYB transcription factor known as *LvMYB5*, which directly bound to the promoter of *LvANS* to enhance the accumulation of anthocyanins in lily petals [16]. Our investigation uncovered a downregulation of the *LvMYB5* gene after *LvBBX24* silencing, suggesting that *LvMYB5* may be a target of LvBBX24. Moreover, *LvMYB5* exhibited an expression pattern similar to *LvBBX24* following light treatment (Fig. S4, see online supplementary material).

**Figure 3:**
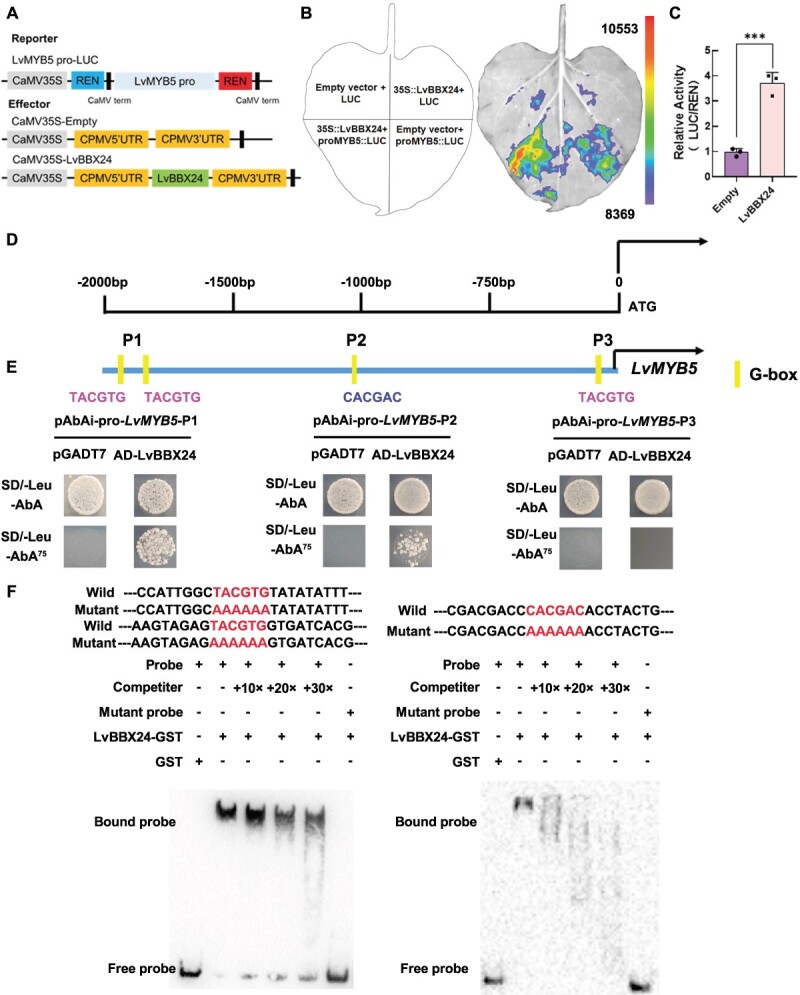
LvBBX24 interacts with the *LvMYB5* promoter to activate its transcription. **A** The Dual-luciferase reporter assay (LUC) reporter vector containing the *LvMYB5* promoter and the effector vector containing LvBBX24 are depicted in a schematic diagram. **B** The transient LUC imaging assay demonstrates that LvBBX24 activates the transcription of the *LvMYB5*pro: LUC reporter gene. Representative images of LUC activity in *Nicotiana benthamiana* leaves were captured 48 hours after infection. **C** The LUC assay shows the promoter activity expressed as the ratio of LUC to 35S::Renilla (REN). The data represents the means ± SD of three replicate measurements. Significant differences were determined using Student’s *t* test (^***^P < 0.001). **D** The specific location of the G-BOX binding site on the LvMYB5 promoter. **E** The yeast one-hybrid assay (Y1H) analysis reveals the binding of LvBBX24 to the *LvMYB5* promoter. **F** EMSA analysis revealed that LvBBX24 specifically binds to the *LvMYB5* promoter fragment that contains the G-box motif. Unlabeled probes were utilized in competition assays, while mutated probes *LvMYB5*-P1 (TACGTG to AAAAAA) and *LvMYB5*-P2 (CACGAC to AAAAAA) were employed to confirm the specificity of the binding site. In this context, ‘−’ denotes absence and ‘+’ denotes presence.

To further characterize the mechanism behind anthocyanin accumulation mediated by LvBBX24, we performed an analysis of the *LvMYB5* promoter, identifying a 2230 bp sequence through chromosome walking. To assess the activation of the *LvMYB5* promoter, we constructed the *LvMYB5*-pro-LUC reporter vector (Fig. 3A) and used the 35S promoter to drive the expression of *LvBBX24* in tobacco leaves. The findings, as depicted in Fig. 3B, revealed increased fluorescence intensity, demonstrating that LvBBX24 activated the *LvMYB5* promoter. The enzyme activity (Fig. 3C) further supported this finding. Our analysis uncovered the presence of G-box binding sites (TACGTA/CACGAC) at positions −1926, −1842, −1062, and −128 (Fig. 3D). These G-box binding sites serve as potential binding sites for BBXs [33]. Subsequently, we digested and cloned the promoter to create yeast one-hybrid system vectors called P1, P2, and P3. Our experimental results demonstrated that LvBBX24 can indeed bind to the P1/P2 promoter region (Fig. 3E). Subsequent electrophoretic mobility shift assay (EMSA) experiments were performed to validate LvBBX24’s binding ability to the G-box binding site on *LvMYB5-pro*. The results revealed successful binding of LvBBX24-GST to the G-box at both P1 and P2 positions, which is evident from the observed probe shift above the free probe. The strength of the binding gradually diminished with an increasing number of cold probes. Notably, upon the introduction of the mutant probe, the shifted probe was no longer detectable (Fig. 3F). These observations robustly suggest that LvBBX24 binds to the *LvMYB5* promoter, influencing anthocyanin biosynthesis in lily petals. Based on the silenced of *LvBBX24*, we enhanced the expression of *LvMYB5* and obtained compelling data indicating the influence of LvBBX24 on the regulatory role of *LvMYB5* in anthocyanin accumulation (Fig. S5A, see online supplementary material).

### LvBBX24 can interact with LvbZIP44

**Figure 4:**
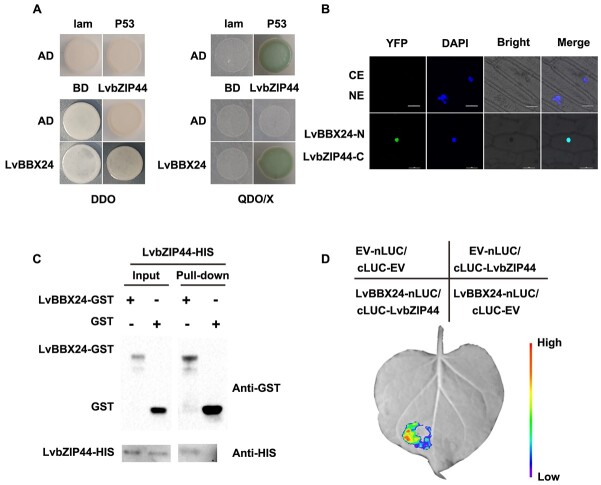
LvBBX24 interacts with LvbZIP44. **A** The interaction between LvBBX24 and LvbZIP44 was tested using a Y2H assay. Positive controls pGADT7-T and pGBKT7–53 were used. The interaction was observed in DDO, SD/−Trp/−Leu medium, and QDO/X, SD/−Trp/−Leu/−Ade/−His medium with X-α-gal. **B** The physical association between LvBBX24 and LvbZIP44 was further validated using a BiFC assay. The bars in the image represent 20 μm. **C** The interaction between LvBBX24 and LvbZIP44 was also confirmed using a pull-down assay. *Escherichia coli* expressed GST or LvBBX24-GST protein and LvbZIP44-HIS fusion protein were incubated with GST affinity resin. Western blot analysis was performed using HIS-tag and GST-tag antibodies. **D** The interaction between LvBBX24 and LvbZIP44 was visualized *in vivo* using a double LUC image. The bar on the right side of the image represents the intensity of the luciferase signal.

Prior studies have demonstrated the ability of BBXs to regulate downstream gene expression through interactions with other transcription factors [34]. In this study, we investigated the regulatory mechanism of LvBBX24 in anthocyanin synthesis. To achieve this, we developed LvBBX24-pGADT7 and employed a screening library for yeast two-hybrid analysis (Y2H). Our results revealed the successful identification of a transcription factor known as LvbZIP44 (Fig. S3B, E, F, see online supplementary material), made up of 121 amino acids, which interacts with LvBBX24 (Fig. 4A). To delve deeper into this interaction, we purified LvBBX24 expressed in a prokaryotic system and performed pull-down experiments using GST and LvbZIP44-HIS. The results of the pull-down experiment confirmed the *in vitro* interaction between LvBBX24 and LvbZIP44 (Fig. 4C). In addition, we employed bimolecular fluorescence complementation (BiFC assay) and Firefly luciferase complementation imaging (LCI) experiments to generate further evidence for the *in vivo* interaction between LvBBX24 and LvbZIP44 (Fig. 4D).

### LvbZIP44 is unresponsive to light but can still play a role in anthocyanin biosynthesis.

**Figure 5:**
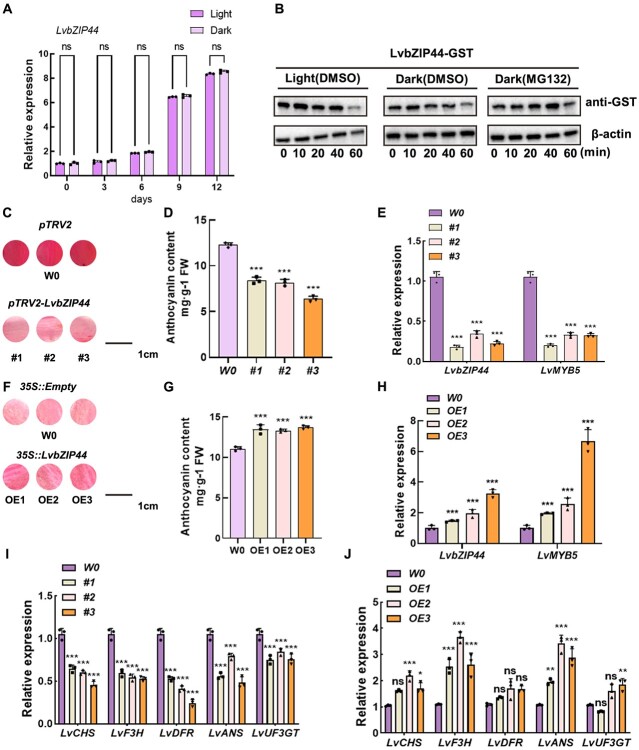
LvbZIP44 affects anthocyanin biosynthesis through a light-independent pathway. **A**  *LvbZIP44* expression pattern was assessed after light treatment. **B**  *In vitro* degradation experiments were performed to analyse the protein level changes of LvbZIP44-GST under different light and dark conditions. Total protein was extracted and treated with either DMSO or MG132. Samples were taken at specific time intervals (0, 10, 20, 40, and 60 minutes) with actin used as the internal control. **C** Accumulation of anthocyanins in lily petals was observed after silencing of *LvbZIP44*. **D**  *LvbZIP44* was silenced to measure anthocyanin content to evaluate the effect. **E** The relative expression levels of *LvbZIP44* and *LvMYB5* genes in silenced lilies. **F** The accumulation of anthocyanins in lily petals was observed after transient overexpression of *LvbZIP44*. **G**  *LvbZIP44* was transiently overexpressed and the anthocyanin content of lily petals was measured to evaluate the effect. **H** Relative expression levels of *LvbZIP44* and *LvMYB5* in lily petals overexpressing *LvbZIP44*. **I** Expression levels of structural genes in silenced *LvbZIP44* lily petals. **J** Analysis of the expression levels of structural genes in lily petals overexpressing *LvbZIP44.* Data in (A–J) are means ± SD (*n* = 3). Statistical significance in A and D–G was analysed using Student’s *t*-test (^***^P < 0.001; n.s., no significant difference).

To elucidate LvbZIP44’s role in lily anthocyanin biosynthesis, we initially explored its participation in light-responsive anthocyanin production. Specifically, we evaluated *LvbZIP44* activity in lily petals exposed to light and dark conditions over 12 days. Expression levels were quantified (Fig. 5A). Intriguingly, we noted a gradual rise in *LvbZIP44* expression over time, with no significant variation in relative expression between light and dark treatments. These observations suggest that *LvbZIP44* expression remains unaffected by light exposure. Subsequently, we investigated the *in vitro* degradation of LvbZIP44 and observed no discernible differences in protein abundance under different light conditions. Furthermore, the addition of MG132 did not significantly alter LvbZIP44’s protein content, prompting further investigation (Fig. 5B). To examine the potential involvement of LvbZIP44 in the anthocyanin biosynthetic pathway of lily petals, we performed a brief silencing experiment on *LvbZIP44* (Fig. 5C). The results suggested that the anthocyanin content of TRV-*LvbZIP44* was significantly reduced compared to the TRV control (Fig. 5D). RT-qPCR analysis of genes linked to the anthocyanin synthesis pathway uncovered a significant down-regulation of *LvMYB5*, *CHS*, *F3H*, *DFR*, *ANS*, and *UF3GT*, among other genes (Fig. 5E and I). Moreover, we conducted a transient overexpression of *LvbZIP44* (Fig. 5F) and identified an up-regulation in the anthocyanin expression of 35S::*LvbZIP44* and *LvMYB5* genes, alongside 35S::Empty (Fig. 5G). The expression levels of *LvMYB5* and the structural genes significantly increased (Fig. 5H and J).

### LvbZIP44 is capable of positively regulating anthocyanin accumulation by binding to the *LvMYB5* promoter

**Figure 6:**
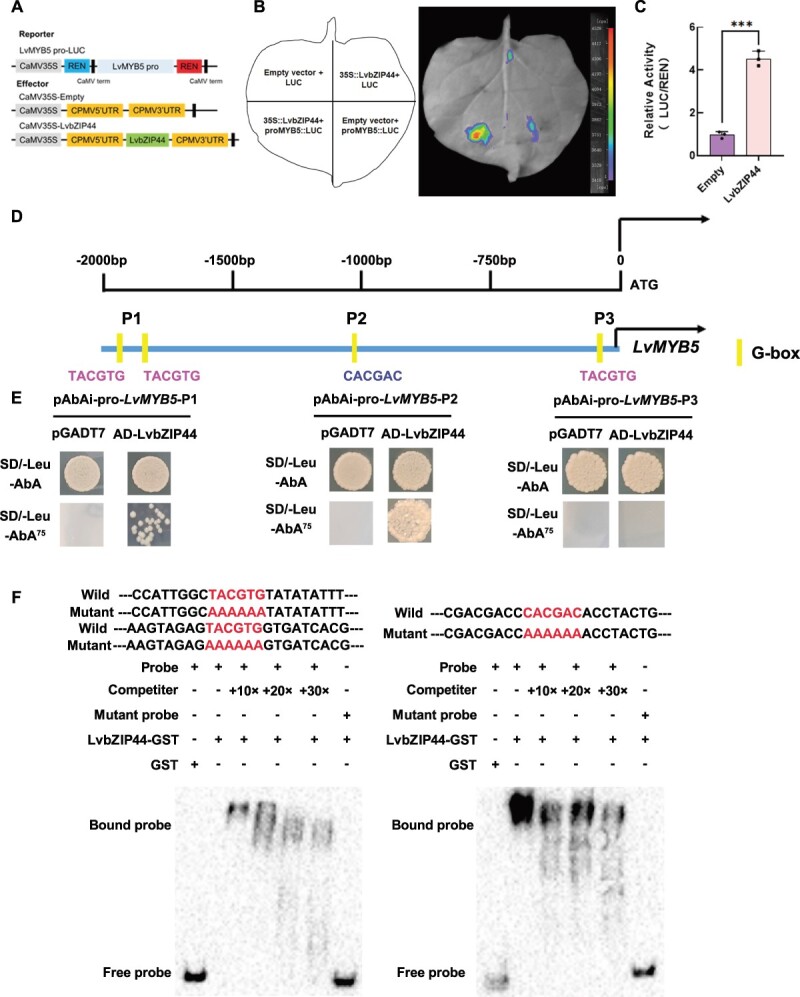
**A** A schematic representation is provided, depicting the LUC reporter vector containing the *LvMYB5* promoter and the effector vector containing LvbZIP44. **B** A transient LUC imaging assay demonstrates that LvbZIP44 activates transcription of the *LvMYB5pro*: LUC reporter gene. Representative images of LUC activity in *Nicotiana benthamiana* leaves captured 48 hours after infection are shown. **C** A dual LUC assay is performed to show the promoter activity expressed as the ratio of LUC to 35S::Renilla (REN). Data represent the mean ± SD of three replicate measurements. Significant differences were determined using Student’s *t* test (***P < 0.001). **D** An illustration of the *LvMYB5* promoter site and its segmentation is provided. **E** Y1H analysis reveals the binding of LvbZIP44 to the *LvMYB5* promoter. **F** EMSA analysis revealed that LvbZIP44 specifically binds to the *LvMYB5* promoter fragment that contains the G-box motif. Unlabeled probes were utilized in competition assays, while mutated probes *LvMYB5*-P1 (TACGTG to AAAAAA) and *LvMYB5*-P2 (CACGAC to AAAAAA) were employed to confirm the specificity of the binding site. In this context, ‘−’ denotes absence and ‘+’ denotes presence.

To assess the influence of LvbZIP44 on anthocyanin accumulation, we examined the relationship between LvbZIP44 and the *LvMYB5* promoter. We generated the *LvMYB5*-pro-LUC reporter gene (Fig. 6A) and co-transformed it alongside 35S::LvbZIP44 into tobacco leaves using *Agrobacterium tumefaciens*. Through dual-luciferase imaging and enzyme activity analysis, we identified that LvbZIP44 significantly activated the *LvMYB5* promoter (Fig. 6B and C). Moreover, yeast one-hybrid analysis revealed that LvbZIP44 was able to bind to the G-BOX binding site on the *LvMYB5* promoter. This was clear from the yeast co-transfected with LvbZIP44-AD, as well as P1 and P2, which were not inhibited by AbA (Fig. 6D and E). EMSA experiments were conducted to confirm the interaction between LvbZIP44 and the *LvMYB5* promoter *in vitro*. The findings indicated that LvbZIP44-GST successfully bound to biotin-labeled *LvMYB5*-P1 and *LvMYB5*-P2. When probes without biotin labeling were introduced, the intensity of the bound band decreased. Furthermore, LvbZIP44-GST was unable to bind to the mutant probe (Fig. 6F). We overexpressed *LvMYB5* on the basis of silencing *LvbZIP44*, and the results also showed evidence that LvBBX24 affects *LvMYB5* in regulating anthocyanin accumulation (Fig. S5B, see online supplementary material).

**Figure 7:**
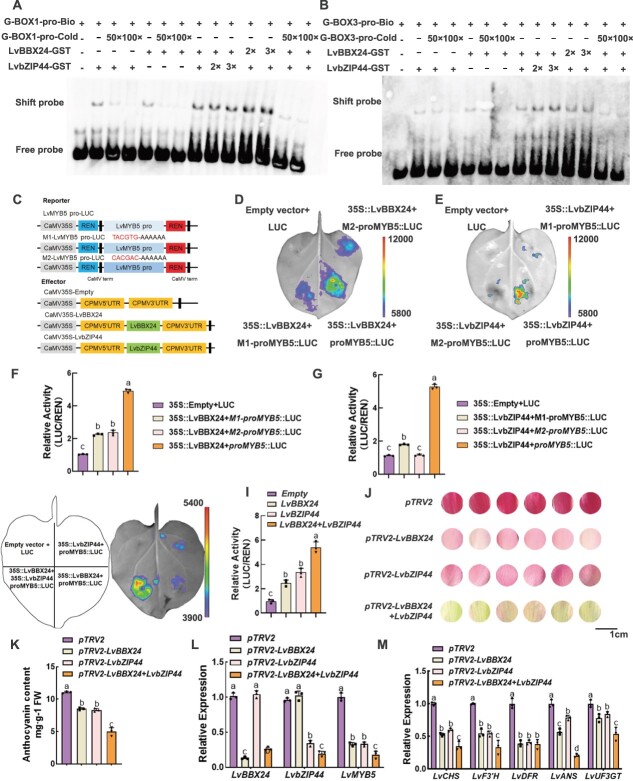
The LvBBX24-LvbZIP44 complex plays a joint role in promoting anthocyanin accumulation. **A** and **B** LvBBX24 and LvbZIP44 bind to the P1-G-box and P2-G-box element of the *LvMYB5* promoter as observed in the EMSA. The probe sequences used are shown. The designations 50× and 100× indicate the use of an excess cold competition probe, while 2× and 3× indicate the use of corresponding multiples of fusion protein. The symbols ‘+’ and ‘−’ indicate the presence and absence of protein-DNA complexes or free probes, respectively. Arrows represent the complexes or probes. **C** A schematic illustrates the LUC reporter vector containing the *LvMYB5* promoter and the effector vector containing LvBBX24 and LvbZIP44. **D** and **E** are dual LUC assays using *LvMYB5*-pro mutant binding sites: D converts the binding site (TACGTA to AAAAAA), E the binding site (CACGAC to AAAAAA). **F** The transient LUC imaging assay demonstrates that LvBBX24 and LvbZIP44, either alone or together, activate the transcription of the *LvMYB5*pro: LUC reporter gene. Representative images of LUC activity in *Nicotiana benthamiana* leaves were captured 48 hours after infection. **G** The dual LUC assay shows the promoter activity expressed as the ratio of LUC to 35S::Renilla (REN). The data represents the mean ± SD of three replicate measurements. Different letters in F–M indicate significant differences by Tukey s-b (K) test with *P* < 0.05. **H** The individual silencing of *LvBBX24* or *LvbZIP44*, as well as the simultaneous silencing of *LvBBX24* and *LvbZIP44*, was performed. This was done to investigate the accumulation of anthocyanins in lily petals. **I** The anthocyanin content was determined to assess the level of accumulation. **J** Expression levels of *LvBBX24*, *LvbZIP44*, and *LvMYB5* in silenced *LvBBX24*, *LvbZIP44* and both *LvBBX24* and *LvbZIP44* lily petals. **K** The expression of structural genes in silenced *LvBBX24*, *LvbZIP44* and both *LvBBX24* and *LvbZIP44* lily petals.

### The LvBBX24-LvbZIP44 complex co-promotes anthocyanin accumulation

In this study, we sought to characterize the influence of the LvBBX24-LvbZIP44 complex on anthocyanin synthesis. We performed an EMSA using LvBBX24 and LvbZIP44 to examine the impact of the LvBBX24-LvbZIP44 complex on the *LvMYB5* promoter. Notably, we added equal amounts of protein to each group to guarantee accurate comparisons.

Our findings indicate two outcomes from EMSA. Firstly, the presence of two shift bands concurrently suggests that, besides the binding of a single LvBBX24 or LvbZIP44 protein to the *LvMYB5* promoter, there is also the binding of the LvBBX24-LvbZIP44 complex to the *LvMYB5* promoter (Fig. S6, see online supplementary material). Secondly, a single shift is observed, which varies with different protein concentrations. In scenarios with an excess probe, when both LvBBX24 and LvbZIP44 proteins are present at the P1 (TACGTA) binding site simultaneously, the shift band appears darker compared to when only one protein is present. Interestingly, an excess of LvBBX24 darkens the shift band, while an excess of LvbZIP44 does not impact the intensity of the shift color. Conversely, at the P2 (CACGAC) binding site, an excess of LvbZIP44 protein results in a darker shift band compared to equal amounts of LvBBX24 and LvbZIP44, even when the latter is twice or five times the amount of LvBBX24. This leads us to hypothesize that P1 serves as the primary binding site for LvBBX24, while P2 is the primary binding site for LvbZIP44 (Fig. 7A–B), the results of the Dual-LUC experiment with mutation sites were consistent with previous findings. Mutating site P1 (TACGTG to AAAAAA) and adding 35S::LvBBX24 + *MYB5-pro*::LUC resulted in a significantly higher signal compared to 35S::LvBBX24 + *M1-MYB5-pro*::LUC. Similarly, mutating site P2 (CACGAC to AAAAAA) and adding 35S::LvbZIP44 + *MYB5-pro*::LUC led to a higher signal than 35S::LvBBX24 + *M2-MYB5-pro*::LUC. These results indicate that the specific mutation site affects the binding ability of LvBBX24 and LvbZIP44 to the *LvMYB5* promoter (Fig. 7C–E). And these results confirm our conjecture that they work by binding to their respective main sites.

Subsequently, the fusion vector containing the 35S promoter was introduced into tobacco leaves using *Agrobacterium*. Specifically, Empty, 35S::LvBBX24, and 35S::LvbZIP44 vectors were transformed into tobacco leaves. The findings indicated that the fluorescence intensity and enzyme activity were significantly higher when LvBBX24 and LvbZIP44 were present in concert, compared to individual treatments (Fig. 7H and I). Additionally, *LvBBX24* and *LvbZIP44* were silenced through virus-induced gene silencing (VIGS) (Fig. 7J). The results demonstrated that the anthocyanin content was significantly lowered when *LvBBX24* and *LvbZIP44* were simultaneously silenced, relative to silencing only one of them (Fig. 7K). Fluorescence quantitative analysis revealed that silencing of *LvBBX24* or *LvbZIP44* alone led to a 0.5-fold decrease in anthocyanin content while silencing both genes simultaneously resulted in a 0.75-fold decrease in anthocyanin content. The expression of *LvMYB5* and the determination of structural gene levels demonstrated that silencing *LvBBX24* or *LvbZIP44* alone had a lesser impact on anthocyanin levels compared to silencing both genes simultaneously (Fig. 7L and M).

## DISCUSSION

### LvBBX24 may operate as a major regulator of light-induced anthocyanin biosynthesis in lily

The influence of light on anthocyanin accumulation has been extensively examined in various plant species [25,35,36]. Previous studies have shown that light promotes anthocyanin accumulation in strawberries and chrysanthemums [35,37]. Similarly, our study affirmed this conclusion in lily (Fig. 1; Fig. S7, see online supplementary material). However, the regulatory mechanism of anthocyanins in lily remains incompletely characterized. Most current research on plant anthocyanins focuses on the MYB-bHLH-WD40 ternary complex, with the R2R3-MYB transcription factor being an essential component [17,38]. Upstream transcription factors have received limited attention. In our study, a light-induced expression protein named as LvBBX24 was discovered in lily.

LvBBX24 binds to the promoter of Lv*MYB5*, a gene that promotes anthocyanin synthesis [16], upregulating *LvMYB5* transcription. Overexpression of *LvBBX24* caused increased expression of *LvMYB5* and enhanced anthocyanin accumulation. In contrast, short-term inhibition of *LvBBX24* significantly lowered anthocyanin accumulation in lily petals and downregulated anthocyanin-related genes (Figs 2 and 3). *LvBBX24* was consistently overexpressed in *Arabidopsis*, resulting in an increased accumulation of anthocyanins at the stem base and higher anthocyanin content in seeds compared to wild-type *Arabidopsis*. Additionally, LvBBX24-GFP protein was detected in 35S::LvBBX24 *Arabidopsis*. Gene expression analysis revealed a significant elevation in the expression levels of *AtCHS*, *AtDFR*, *AtANS*, and *AtF3’H* (Fig. S8D–G, see online supplementary material). Overexpression of *LvBBX24* in apple callus augmented anthocyanin accumulation and elevated the expression of apple anthocyanin biosynthetic genes (Fig. S9A–C, see online supplementary material).

LvBBX24 is homologous to *Arabidopsis* BBX24 (Fig. S3A, see online supplementary material). In *Arabidopsis*, BBX24 can function independently or in concert with other proteins to influence photomorphogenesis [39,40]. Our study revealed a potential interaction between LvBBX24 and LvCOP1, suggesting a possible regulatory role in plant growth and development through their interaction (Fig. S7, see online supplementary material). Similarly, *Arabidopsis* BBX24 can interact with COP1 and HY5 during photomorphogenesis [41,42]. In our study, we identified that the expression of *LvBBX24* was induced by light (Fig. 2A) and significantly decreased in darkness (Fig. 2B), aligned with previous findings [43,44]. It is hypothesized that LvBBX24 may experience degradation following interaction with LvCOP1, although further characterization is required to verify the specific mechanism. Our experimental results indicate that LvBBX24 functions as a positive transcription factor, enhancing the transcription of *LvMYB5* in response to light. In addition, based on the current protein abundance of LvBBX24 in darkness, we hypothesize that LvBBX24 shares similarities with the BBX family in *Arabidopsis*, and similarly, LvBBX24 is likely degraded in the absence of light, losing its activating activity on *LvMYB5*. *Arabidopsis* plants overexpressing *LvBBX24* were subjected to light treatment, resulting in induced anthocyanin accumulation. The LvBBX24-GFP protein abundance was observed to degrade rapidly in the absence of light, while the expression levels of structural genes were upregulated in response to light (Fig. S8A–C, see online supplementary material). Overall, our experiments provide compelling evidence that LvBBX24 functions as a light-responsive BBX transcription factor.

### LvbZIP44 can act alone to regulate anthocyanins, which is the possible reason for the accumulation of anthocyanins in lilies under dark conditions.

In transcription factor regulation, the interaction between multiple transcription factors plays a crucial role in regulating changes in downstream genes. Our experiments uncovered that LvBBX24 and LvbZIP44 synergistically influence the regulation of anthocyanins, as illustrated in Fig. 4A–D. This effect is similar to the role of HY5 in *Arabidopsis* [45]. Furthermore, our study indicated that the presence of LvBBX24 and LvbZIP44 simultaneously impacts the activity of the *LvMYB5* promoter and promotes transcription of *LvMYB5*, causing an increase in anthocyanin levels. Further studies found that LvbZIP44 functions independently without being regulated by light (Fig. 5A and B; Fig. S7A, see online supplementary material). To examine this further, we performed transient overexpression and silencing experiments on *LvbZIP44*, as illustrated in Fig. 5C and F. Notably, we observed that the expression level of the critical anthocyanin-regulated gene *LvMYB5* mirrored the expression changes of *LvbZIP44*, as illustrated in Fig. 5D and G. Silencing of *LvbZIP44* resulted in a reduction in anthocyanin content (Fig. 5E), while overexpression of *LvbZIP44* enhanced anthocyanin accumulation (Fig. 5H). The bZIP transcription factor family plays a crucial role in diverse aspects of plant growth and development, encompassing resistance to stress and participation in cell signal transduction. Recent studies have demonstrated that bZIP transcription factors may be involved in pathways influencing anthocyanin biosynthesis. In the research of apple, the bZIP transcription factor ABI5 has been identified as a regulator of ABA-induced anthocyanin biosynthesis via its interaction with the MYB1-bHLH3 complex [46]. In addition, a new type of transcription factor known as MdbZIP23 indirectly interacts with MdNAC1, promoting anthocyanin biosynthesis. However, it has not been determined whether MdbZIP23 directly regulates anthocyanin accumulation [47]. Our study focused on the transcription factor LvbZIP44 and its function in regulating anthocyanins. We uncovered that LvbZIP44 can directly bind to the promoter of *LvMYB5*, exerting control over anthocyanin levels (Fig. 6A–F). Overexpression of *LvbZIP44* in the ‘Orin’ apple callus led to a significant enhancement in anthocyanin content relative to empty and wild-type apples. The expression levels of structural genes mirrored this enhancement (Fig. S9A–C, see online supplementary material).

Given the expansive diversity within the bZIP family, gene functions are likely differentiated, and specific regulatory mechanisms linked to anthocyanins must be unraveled. According to our experimental findings, LvbZIP44 acts as a positive regulator of anthocyanin accumulation in lilies through interaction with the promoter of *LvMYB5*. Moreover, given that LvBBX24 is regulated by light and undergoes rapid degradation in the absence of light, our investigation revealed that the expression level of *LvbZIP44* remains unaltered by light exposure, with no discernible change in protein abundance observed in the dark (Fig. 5A and B; Fig. S7A, see online supplementary material). Notably, LvCOP1, known for its pivotal role in protein degradation under dark conditions, does not interact with LvbZIP44 (Fig. S10, see online supplementary material). We speculate that LvbZIP44 is critical in dark conditions and influences anthocyanin accumulation via its interaction with the promoter of *LvMYB5*.

### LvBBX24 and LvbZIP44 may interact to create a circular fold on the LvMYB5 promoter, altering its DNA structure and collectively influencing the anthocyanin accumulation in lily petals

In our study, we determined that both LvBBX24 and LvbZIP44 can enhance the production of lily anthocyanins. Moreover, both proteins can interact with the G-box binding element in the *LvMYB5* promoter region, causing the up-regulation of *LvMYB5* transcription. To explore this relationship, we performed EMSA experiments. *In vitro*, both LvBBX24 and LvbZIP44 could independently bind to the G-BOX site within P1. However, when both proteins were present collectively, no competition was observed. Instead, the intensity of the shifted band increased alongside a higher concentration of LvBBX24, suggesting that LvBBX24 may play a major role in the first G-box of *LvMYB5* (Fig. 6A). The EMSA findings for binding site P2 demonstrated that LvBBX24 and LvbZIP44 can bind individually to G-boxes. However, unlike P1, a different scenario was observed at this binding site. When the LvbZIP44-GST fusion protein concentration was elevated, the intensity of the shifted band was higher compared to the case in which equal amounts of LvBBX24 and LvbZIP44 were present simultaneously. No competitive inhibitory function was identified (Fig. 6B). EMSA was utilized to validate the binding affinity of the LvBBX24 + LvbZIP44 complex to the LvMYB promoter. Findings indicated that the concurrent introduction of LvBBX24-GST and LvbZIP44-GST yielded two distinct shift bands above the free probe. Conversely, when LvBBX24-GST was introduced alone, only one shift band observed for LvbZIP44-GST binding to the *LvMYB5* promoter (Fig. S10, see online supplementary material). Multiple experiments have demonstrated the rarity of two shift bands, leading us to hypothesize that LvBBX24 and LvbZIP44 regulate the expression of *LvMYB5* by binding to the main site *in vitro*. Subsequent *in vivo* validation supported our hypothesis, revealing that LvBBX24 predominantly binds to P1 and LvbZIP44 primarily binds to P2 (Fig. 7D–G). In research on carnations, the presence of DcHB30 and DcWRKY75, two interacting proteins, can impact the activity of their respective downstream binding. In particular, an increase in the presence of DcHB30 and DcWRKY75 alone leads to a reduction in the binding ability of these proteins to downstream targets [48].

Transcription factor cooperativity is critical in eukaryotic transcriptional regulation, involving multiple mechanisms including protein–protein/or protein–DNA interactions [49]. Prior studies have shown that when interacting transcription factors bind collectively, they form a helical structure and bind to their specific binding sites [50]. In addition, transcription factors can induce conformational changes in DNA, enabling improved binding with transcription factors [51]. Research on potatoes has revealed that the uneven distribution of basic residues in the bZIP-like domain of the StTGA2.1-sttga2.2 heterodimer significantly alters the DNA conformation near its binding site. This altered DNA conformation plays a key role in activating promoter activity [52]. Furthermore, the interaction between two NPR1 proteins links two AtTGA3 homodimers that are attached to distinct DNA-binding motifs, creating an AtTGA32-NPR12 complex that acts as an enhancer for AtTGA32, ultimately leading to transcription activation [53]. These studies have demonstrated that the interaction of transcription factors can induce varying degrees of DNA conformational changes, thereby facilitating transcription.

According to these findings, we speculate that LvBBX24, differing by approximately 1000 bp from the two binding sites, may also generate multiple complexes with LvbZIP44 to bind to the promoter of *LvMYB5*, enhancing the transcription of *LvMYB5*. The interaction between LvBBX24 and LvbZIP44 potentially alters the conformation of the DNA region in the *LvMYB5* promoter, causing improved recognition and binding of the two transcription factors when present simultaneously. However, the specific mechanism of action remains to be further studied.

**Figure 8:**
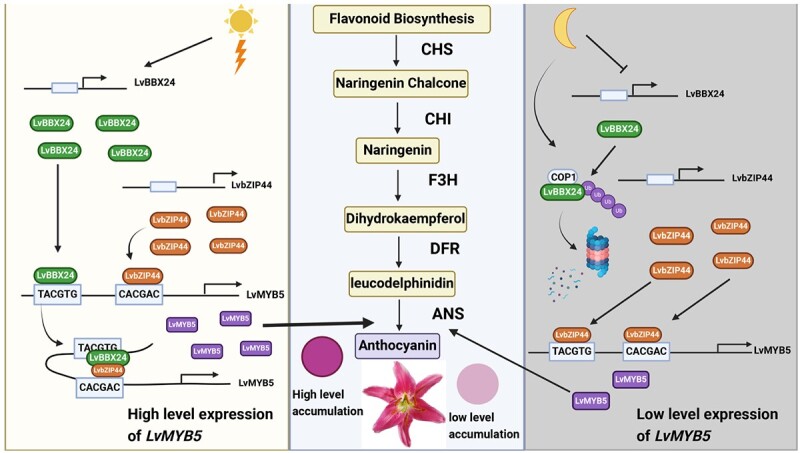
This figure illustrates the influence of LvBBX24 and LvbZIP44 on anthocyanin synthesis. Acting as individual transcription factors, LvBBX24 and LvbZIP44 can modulate anthocyanin accumulation by binding to the LvMYB5 promoter. LvBBX24, a member of the typical BBX protein family, is upregulated in response to light. Exposure of lily petals to daylight increases LvBBX24 expression, thereby enhancing *LvMYB5* transcription and promoting anthocyanin accumulation. Moreover, elevated LvBBX24 levels enable complex formation with LvbZIP44, binding to the primary G-box site to change the configuration of the promoter. Subsequently, LvBBX24 undergoes rapid degradation, while LvbZIP44 assumes a pivotal role in enhancing anthocyanin accumulation by binding to the *LvMYB5* promoter. In summary, our study unveils novel functionalities of LvBBX24 and LvbZIP44 in anthocyanin synthesis, enriching our understanding of the regulatory network governing anthocyanin biosynthesis.

To examine the role of LvBBX24 and LvbZIP44 in anthocyanin accumulation, we performed transient silencing experiments using *Agrobacterium*-mediated transformation. Our results indicated that silencing either *LvBBX24* or *LvbZIP44* in isolation led to reduced anthocyanin content, gene expression, and anthocyanin accumulation relative to the control (TRV null). However, the simultaneous silencing of both genes caused a significant reduction in anthocyanin accumulation in the petals, even lower than in TRV-*LvBBX24* and TRV-*LvbZIP44* plants. Our findings indicate that the LvBBX24-LvbZIP44 complex has its own binding site, playing a pivotal role. During the day, LvBBX24 binds to the P1 binding site, while LvbZIP44 binds to the P2 binding site. The interaction between LvBBX24 and LvbZIP44 in the body affects the promoter folding of *LvMYB5*, leading to joint regulation by the LvBBX24-LvbZIP44 complex. This ultimately enhances the transcription of *LvMYB5*, promoting anthocyanin accumulation in response to light. Conversely, in dark conditions, LvbZIP44 takes on a prominent role due to the rapid degradation of the LvBBX24 protein. It binds to the *LvMYB5* promoter, promoting the accumulation of anthocyanins. While other environmental factors may also modulate the stability of LvBBX24 protein, our study underscores the significant role of LvbZIP44 in regulating anthocyanin accumulation (Fig. 8).

## Materials and methods

### Plant materials and growth conditions

In this study, we utilized Oriental hybrid lily (cultivar ‘Viviana’) as our experimental plant. The lilies were grown in a controlled greenhouse environment at Shenyang Agricultural University, with temperatures ranging from 2–25°C throughout the year 2020. For our *Arabidopsis* experiments, the seeds were germinated in MS medium and after 10 days the seedlings were transferred to 10 cm pots filled with a mixture of rose stone and turf soil. These pots were also placed in the same greenhouse, maintaining day/night temperatures of 22°C/16°C (light intensity: 300–600 μmol·m-2·s-1), and no additional light was provided for any of the treatments. The experiment commenced at the S1 stage, occurring 20 days after the appearance of flower buds, with both bagging and non-bagging treatments. Bagging treatment entailed 24 hours of darkness, while the non-bagging treatment involved 16 hours of light and 8 hours of darkness. Samples were collected at 9 a.m. on days 0, 3, 6, 9 and 12. The light treatment (16 hours of light/8 hours of dark) involved 5-day etiolated *Arabidopsis* seedlings, with illumination initiated on the 5th day and sampling conducted on the 20th day. After successful overexpression, ‘Orin’ apple callus was subcultured in darkness for one month, followed by light treatment (16 hours of light/8 hours of dark). Anthocyanins and RNA were then extracted 12 days later.

### Total RNA extraction, gene cloning, and RT-qPCR assays

Genomic DNA and total RNA extraction were extracted from Lily by EASYspin Plus Plant RNA Kit Aidlab Biotechnologies Co., Ltd (Beijing, China) and cDNA synthesis was carried out according to the steps of MonScriptTM RTIII All-in-One Mix kitMonad Biotech Co., Ltd (Suzhou, China). Primers were designed through Primer Premier 6.0 and submitted to sangon biotechnology for synthesis.

Real-time quantitative PCR (RT-qPCR) was performed as described in MonAmp™ ChemoHS qPCR Mix (Monad Biotech) and the experiment was performed according to its experimental instructions.

### Acquisition of *LvMYB5* promoter

The method of TaKaRa’s Genome Walking kit was used to design specific nested primers *LvMYB5*-SP1, *LvMYB5*-SP2, and *LvMYB5*-SP3 based on the sequence of the *LvMYB5* gene coding region. The primer sequences can be found in Supplementary Table 1. In the first round of amplification, AP1/AP2/AP3/AP4, which are the four degenerate primers included in the kit, along with *LvMYB5*-SP1, were used with lily genomic DNA as a template. The reaction system for this round was 50 μL. The product of the first round of PCR reaction was then used as a template for the second round of amplification, using AP1/AP2/AP3/AP4 and *LvMYB5*-SP2 separately. The reaction system for this round was also 50 μL. Similarly, the product of the second round of PCR reaction was used as a template for the third round of amplification, using AP1/AP2/AP3/AP4 and *LvMYB5*-SP3. The reaction system for the third round was 50 μL. The amplification products of the second and third rounds were separated and detected by 1.0% agarose gel electrophoresis. Samples with specific bands were selected for sequencing by Beijing Qingke Biotechnology Co., Ltd.

### Cloning of the *LvBBX24*and *LvbZIP44* genes and the promoters of *LvMYB5*

The full-length sequences of *LvBBX24*, *LvbZIP44* were cloned from the cDNA of oriental hybrid lily (cultivar ‘Viviana’).

The promoter sequences of *LvMYB5* (2230 bp) were also cloned from the DNA of oriental hybrid lily (cultivar ‘Viviana’). The primers used are shown in Supplementary Table 1 (see online supplementary material).

### Subcellular localization

The coding sequences of *LvBBX24* and *LvbZIP44* without a stop codon were cloned into the BamHI and SalI sites of pCAMBIA1300 to generate LvBBX24-GFP and LvbZIP44-GFP fusion constructs driven by the 35S promoter. Subsequently, the plasmid was transformed into *A. tumefaciens* strain GV3101. The *A. tumefaciens* line carrying the vector was then independently infiltrated into the leaves of tobacco. Finally, fluorescence was observed using confocal laser scanning microscopy (A1, Nikon, Tokyo, Japan).

### Determination of anthocyanin content

To begin the experiment, take 1.0 g of the sample and place it in methanol containing 1% HCl. Let it sit overnight at a temperature of 4°C in a dark environment. The following day, use 400 μL of chloroform to eliminate any chlorophyll present. After centrifuging the mixture at a speed of 14 000 *g* for 5 minutes, measure the absorbance values at 530 nm and 657 nm. To calculate the final result, use the formula A530–0.33 × A657 divided by the fresh weight of the sample. Repeat this process three times for each sample. Statistical significance is indicated as follows: (^*^P < 0.05, ^**^P < 0.01, ^***^P < 0.001).

### Y1H

We integrated the *LvMYB5* promoter sequence into the pABAi vector. Recombinant plasmids were linearized with Bstb1 endonuclease and transformed into Y1H yeast cells, grown on selective medium lacking Ura. The optimal concentration of Aureobasidin A (AbA) for inhibiting autoactivation was determined. In addition, yeast competent cells containing the *LvMYB5* promoter were prepared. After adding LvBBX24-pGADT7 and LvbZIP44- pGADT7 and pGADT7, respectively, a set of treatments involving heat shock and ice bath were carried out. Subsequently, positive clones were chosen on a solid medium deprived of -Leu, which contained the ideal concentration of AbA on the plate. Ascertain the AbA concentration that is most effective in reducing background leakage on media lacking -Trp and -His. Interactions were examined through growth patterns on media without -Trp, -His, and -Leu, but supplemented with AbA.

### EMSA

The Rosetta (DE3) strain was utilized in this study for expressing the GST-LvBBX24 and LvbZIP44 recombinant constructs. The strain was induced with 0.2 mM IPTG at 16°C for 16 hours. The purification of the GST tag and the fusion proteins GST-LvBBX24 and LvbZIP44 was carried out using the Beyotime GST tag protein purification kit (P2262) and the BeyoGold GST tag purification resin, following the manufacturer’s instructions. To investigate the binding of the LvBBX24 and LvbZIP44 fusion proteins to the G-BOX motif in the *LvMYB5* promoter, a probe containing the motif was synthesized and labeled with biotin at the 5′ end using the EMSA Probe Biotin Labeling Kit from Beyotime Biotechnology Institute. EMSA experiments were performed using the Chemiluminescence EMSA Kit provided by Beyotime. The labeled probes or unlabeled oligonucleotides were incubated with the GST-LvBBX24 and LvbZIP44 fusion proteins in a mixture, followed by incubation at 23°C for 30 min. The resulting mixture was then separated on a 6% polyacrylamide gel and visualized using a chemiluminescent imaging system from Bio-Rad Laboratories, Inc.

### Dual-luciferase reporter assay

The luc experiment was carried out as described in the article [16]. The 2230 bp promoter (*MYB5pro* and mutant *MYB5-pro*) of the *LvMYB5* gene was isolated from the lily petal DNA library using gene-specific primers (Supplementary Table 1, see online supplementary material), The M1-pro-MYB5 sequence changes TACGTG to AAAAAA, while the M2-pro-MYB5 sequence changes CACGAC to AAAAAA, and we cloned the full-length *LvMYB5*pro into the BamhI and HindIII sites of the pGreenII 0800-Luc vector, respectively. LvBBX24 or LvbZIP44 constructs were mixed with promoter sequence constructs at a ratio of 1:9 (v/v), and then injected into young leaves of tobacco for transient co-transformation expression analysis. The activity ratio of firefly luciferase and Renilla luciferase was tested using a dual-luciferase reporter assay system (E1910, Promega, USA) following the manufacturer’s instructions. The experiment was performed with three biological replicates, and each biological replicate included three technical replicates.

### Y2H

Protein–protein interaction was performed by Y2H experiment to study whether LvBBX24 protein can bind to LvbZIP44, pGADT7 and pGBKT7-lam vectors were used as negative controls, pGADT7 and pGBKT7-P53 vectors were used as positive controls, yeast carrying LvbZIP44-BD + LvBBX24-AD Grow on SD/−Trp/−Leu and SD/−Trp/−Leu/-His/−Ade + X-α-Gal media.

### LCI

The CDS of LvBBX24 and LvbZIP44 were cloned into the N terminus of luciferase (LUC) in the pCAMBIA-nLUC vector to generate LvBBX24-nLUC and LvbZIP44-nLUC constructs, respectively. Similarly, the CDS of LvBBX24 and LvbZIP44 were cloned into the C terminus of luciferase (LUC) in the pCAMBIA-cLUC vector to generate LvBBX24-cLUC and LvbZIP44-cLUC constructs, respectively. Agrobacterium strain GV3101 (pSoup) carrying nLUC and cLUC recombinant plasmids was mixed and co-transfected into the leaves of *Nicotiana benthamiana*. Forty-eight hours after infiltration, 1 mmol/L D-luciferin potassium substrate was injected into *N. benthamiana* leaves, and the luminescence intensity was detected by using a dual-luciferase reporter gene detection system (NightOWL II LB983; Berthold).

### BiFC

The cDNAs of LvBBX24 and LvbZIP44 were cloned into 35S-SPYNE and 35S-SPYCE vectors, resulting in the creation of LvBBX24-NE, LvbZIP44-1NE, LvBBX24-CE, and LvbZIP44-CE vectors, respectively. These vectors were then transformed into Agrobacterium GV3101 and combined with an Empty vector mix. Different combinations of these vectors were used to infect tobacco leave*s*. After three days of protection from light, the samples were observed using a laser confocal microscope (A1, Nikon, Tokyo, Japan). Additionally, DAPI staining was used to mark the nucleus.

### GST-pull down

Full-length LvBBX24 and LvbZIP44 cDNA were inserted into PGEX-6p and pET 30a vectors, and recombinant GST, LvBBX24-GST, and LvbZIP44-HIS were produced in *Escherichia coli* strain Rosetta. GST tag purification resin (Beyotime, P2262) and HIS tag purification resin (Beyotime, P2226) were used for purification. LvBBX24-GST and LvbZIP44-HIS, GST and LvbZIP44-HIS were mixed, respectively, at 4°C for 2 hours, GST resin added in an ice bath for 2 hours and then eluted, then sampled input and pull down, respectively, adding loading buffer for SDS electrophoresis, Western blotting using anti-GST and anti-HIS antibodies.

### VIGS

For VIGS experiments, we selected 200–300 bp sequences in the cds region and connected them to the pTRV2 vector to construct pTRV2-LvBBX24 and pTRV2-LvbZIP44 recombinant vectors. To administer the injections, we directly injected 100 μL of a solution containing either pTRV2-LvBBX24, pTRV2-LvbZIP44 construct, or Agrobacterium culture of pTRV2 (control) into the underside of the unstained petals. Each time, we infected ten flower buds, which were then photographed 7 days after injection. Additionally, petal samples were frozen in liquid nitrogen and stored at −80°C. This process was repeated three times for each sample. Statistical significance is indicated as follows: ^*^P < 0.05, ^**^P < 0.01, ^***^P < 0.001.

### Transient overexpression

For overexpression experiments, 100 μL of Agrobacterium culture containing PRI-ON-35S-LvBBX24, PRI-ON-35S-LvbZIP44 construct, or PRI-ON-35S (control) was injected directly into the underside of unpigmented petals, each ten flower buds were infected at a time, and phenotypes were observed 7 days after injection. Petal samples were then frozen in liquid nitrogen and stored at −80°C. This process was repeated three times for each sample. Statistical significance is indicated as follows: ^*^P < 0.05, ^**^P < 0.01, ^***^P < 0.001.

### Protein degradation *in vitro*

A total of 1 ml of degradation reaction solution (25 mM Tris–HCl + 10 mM NaCl +10 mM MgCl2 + 4 mM PMSF +10 mM ATP + 5 mM DTT) was added to 1 g of plant material, centrifuged to get supernatant active protein, 50 μL of recombinant protein LvBBX24-GST was added to the supernatant, and light and dark treatment performed, respectively. Samples were taken at intervals of 10–60 minutes, loading buffer added to the samples for SDS electrophoresis, and GST antibody used for western blotting to detect protein abundance.

### Transcription activation verification

In order to determine the transcriptional activation ability of LvBBX24, the full-length cDNA of LvBBX24 was cloned into the pGBKT7 vector, and the constructed vector was independently transformed into the yeast strain Y2HGold together with the positive control (pGBKT7-p53 + pGADT7-T) and the negative control (pGADT7). Yeast transformation and autoactivation tests were performed as previously described [54].

### Statistical analysis

The asterisks indicate significant differences from three biological replicate experiments by a *t*-test (^*^P < 0.05, ^**^P < 0.01, ^***^P < 0.001). Different letters indicate significant differences (*P* < 0.05) revealed by ANOVA followed by Tukey’s correction.

## Supplementary Material

Web_Material_uhae211

## Data Availability

All relevant data in this study are provided in the article and its supplementary figure files.
