# Supplementary material for: Transcription factors LvBBX24 and LvbZIP44 coordinated anthocyanin accumulation in response to light in lily petals
Source: Hortic Res. 2024 Jul 30;11(10):uhae211. doi: 10.1093/hr/uhae211 (PMC11450212; doi:10.1093/hr/uhae211)
Supplement: Web_Material_uhae211 [file web_material_uhae211.zip › Supporting Figures.docx]

**Supporting Figures**


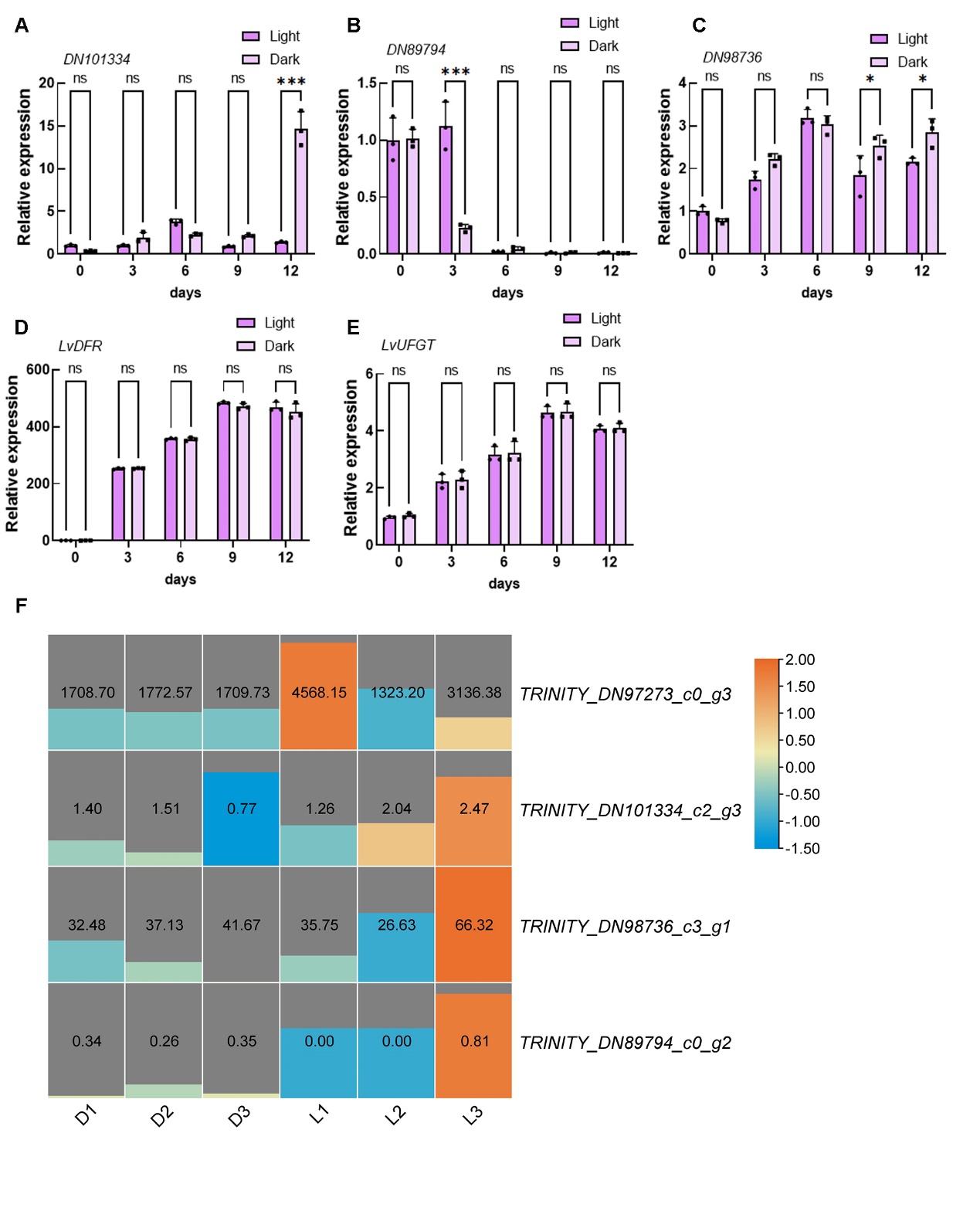


**Figure S1 Relative expression levels of transcriptome B-BOX protein after light treatment. A**, **B** and **C** are the relative expression levels of B-BOX genes code-named ‘*DN101334*’, ‘*DN89794*’ and ‘*DN98736*’ respectively after light treatment. **D** and **E** represent the relative expression levels of structural genes *LvDFR* and *LvUFGT* after light treatment. Data are the means of three biological replicates ± SD. Asterisks indicate significant differences (Student’s t test, *P < 0.05, **P < 0.01, ***P < 0.001) **F** The FPKM value of BBX family genes screened by RNA-seq after light treatment for 24 hours, which involves three repetitions. Dark treatment for 24 hours includes D1, D2, and D3, and light treatment for 24 hours includes L1, L2, and L3.


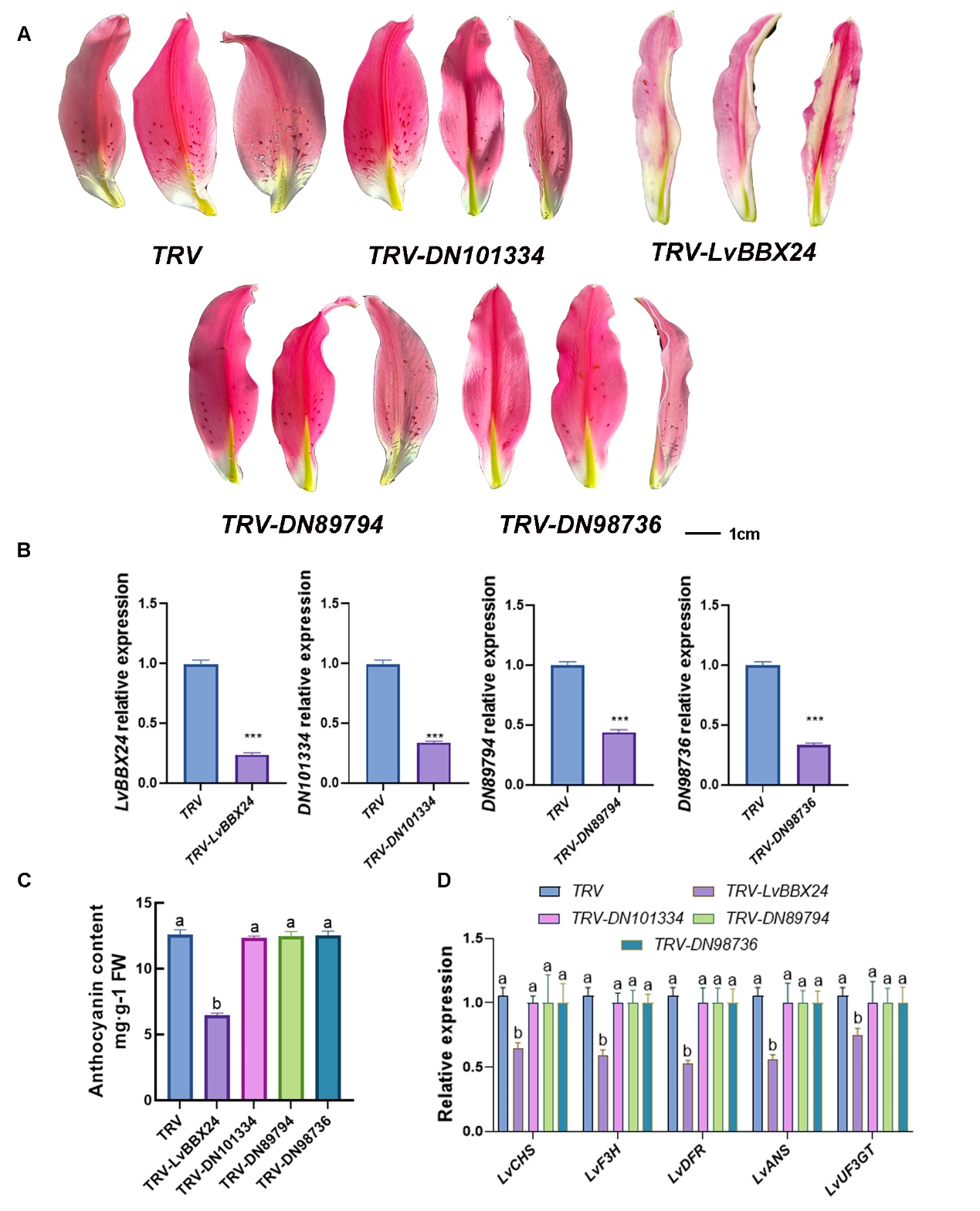


**Figure S2 The BBX genes was silenced by virus induced gene silencing A** Phenotypes of silent TRV, TRV-DN101334, TRV-LvBBX24, TRV-DN89794, and TRV-DN98736 petals. **B** Silencing efficiency of TRV, TRV-DN101334, TRV-LvBBX24, TRV-DN89794, TRV-DN98736. **C** Anthocyanin content in petals of silent TRV, TRV-DN101334, TRV-LvBBX24, TRV-DN89794, and TRV-DN98736. **D** Expression levels of anthocyanin-related structural genes TRV, TRV-DN101334, TRV-LvBBX24, TRV-DN89794, and TRV-DN98736.


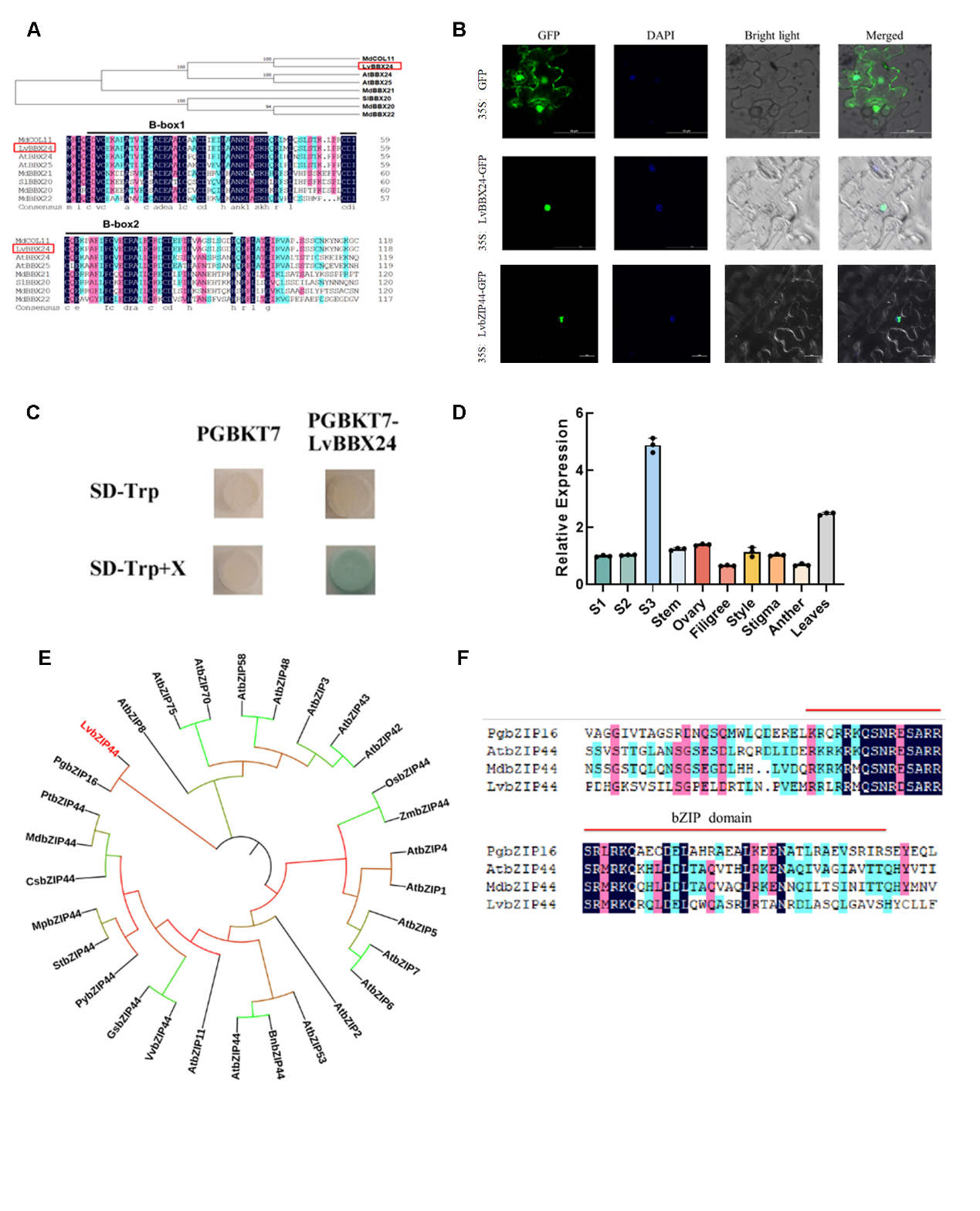


**Figure S3 Evolutionary analysis and expression analysis of LvBBX24.** **A** Phylogenetic analysis of LvBBX24 and selected anthocyanin-related B-BOX from different plants. Lv: **(*Lilium spp.) Asiatic Hybrids*** Sl：*Solanum lycopersicum*（SlBBX20: XM_026028756.1）Md: *Malus domestica* (MdCOL11: XM_029089114.1; MdBBX20: XM_008384103.3; MdBBX21: 26028756.1), At: *Arabidopsis thaliana* (AtBBX24: NP_172094.1; AtBBX25: XP_020883610.1). The highly conserved domains of the B-BOX protein are boxed with black underline marks. **B** Subcellular localization of LvBBX24 and LvbZIP44. **C** Transcriptional activation analysis of LvBBX24.**D** Relative expression of LvBBX24 in different tissues of lily. S1 (20 days after flower buds appear); S2 (30 days after flower buds appear); S3 (40 days after flower buds appear). **E** Phylogenetic analysis of LvbZIP44 with the Arabidopsis bZIP family and selected anthocyanin-related bZIP transcription factors. At: *Arabidopsis thaliana* (AtbZIP1: At5g49450; AtbZIP2:At2g18160; AtbZIP3: At5g15830; AtbZIP4: At1g59530; AtbZIP5: At3g49760; AtbZIP6: At2g22850; AtbZIP7: At4g37730; AtbZIP8: At1g68880; AtbZIP11: At4g34590; AtbZIP42: At3g30530; AtbZIP43: At5g38800; AtbZIP44: At1g75390; AtbZIP48: At2g04038; AtbZIP53 At3g62420; AtbZIP58: At1g13600; AtbZIP70: At5g60830; AtbZIP75 At5g08141); Pg: *Punica granatum* (PgbZIP16: XP_031391917.1); Md: *Malus domestica* (MdbZIP44: XP_008377201.2); Mp: *Mucuna pruriens* (MpbZIP44: RDY09443.1); Zm: *Zea mays* (ZmbZIP44: NP_001167995.1); Py: *Prunus yedoensis var. nudiflora* (PybZIP44: PQQ04057.1); Bn: *Brassica napus* (BnbZIP44: XP_013669840.1); St: *Senna tora* (StbZIP44: KAF7836952.1); Cs: *Cucumis sativus* (CsbZIP44: XP_004152685.1); Gs: *Glycine max* (GsbZIP44: NP_001236565.1); Os: *Oryza sativa Japonica Group* (OsbZIP44: XP_015651067.1); Pt: *Populus trichocarpa* (PtbZIP44: KAI5596869.1); Vv: *Vitis vinifera* (VvbZIP44: XP_002283667.1) **F** Protein sequence alignment of LvbZIP44 with selected anthocyanin-related bZIP transcription factors, with bZIP domains underlined.


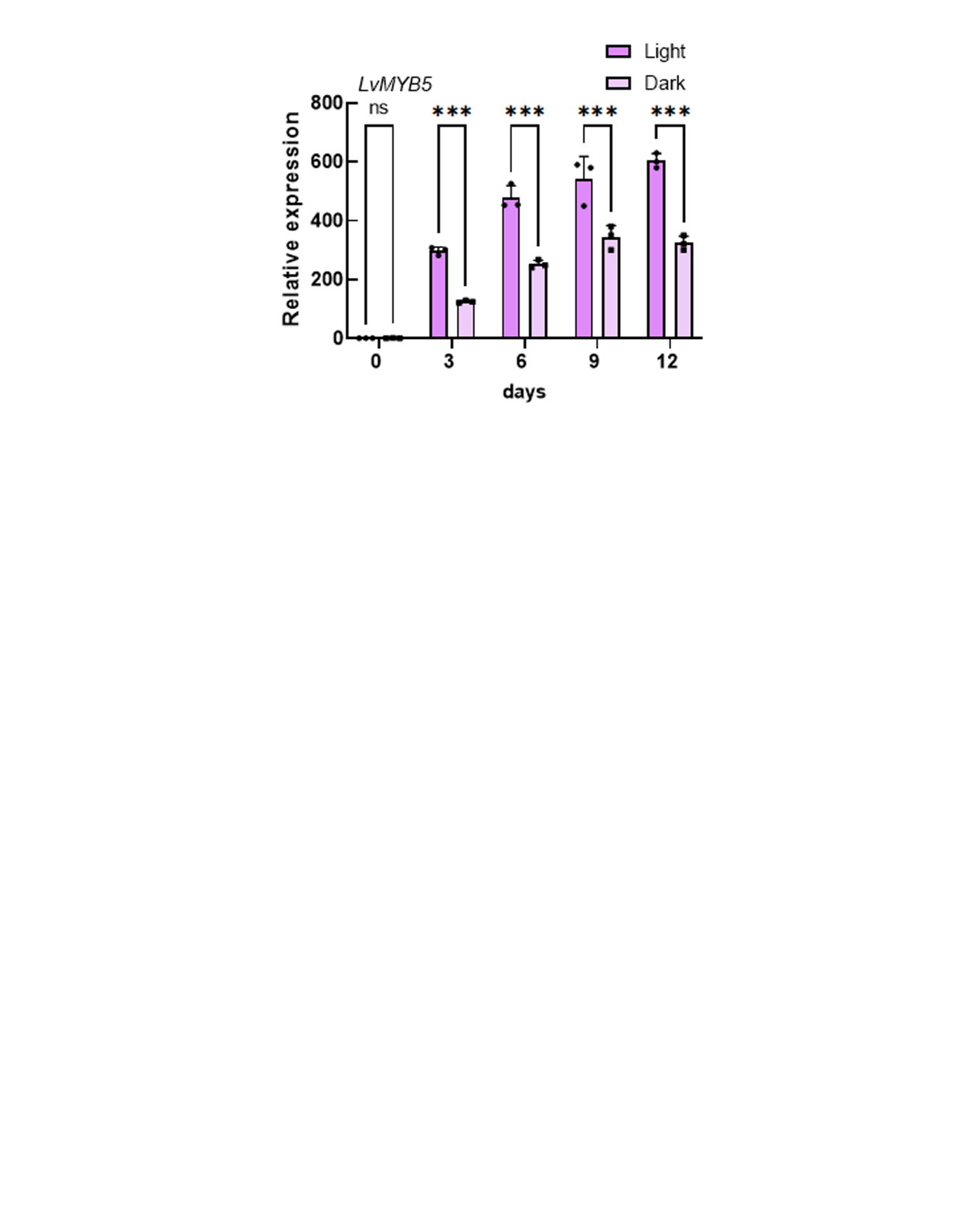


**Figure S4 Expression trend of *LvMYB5* under light treatment.**


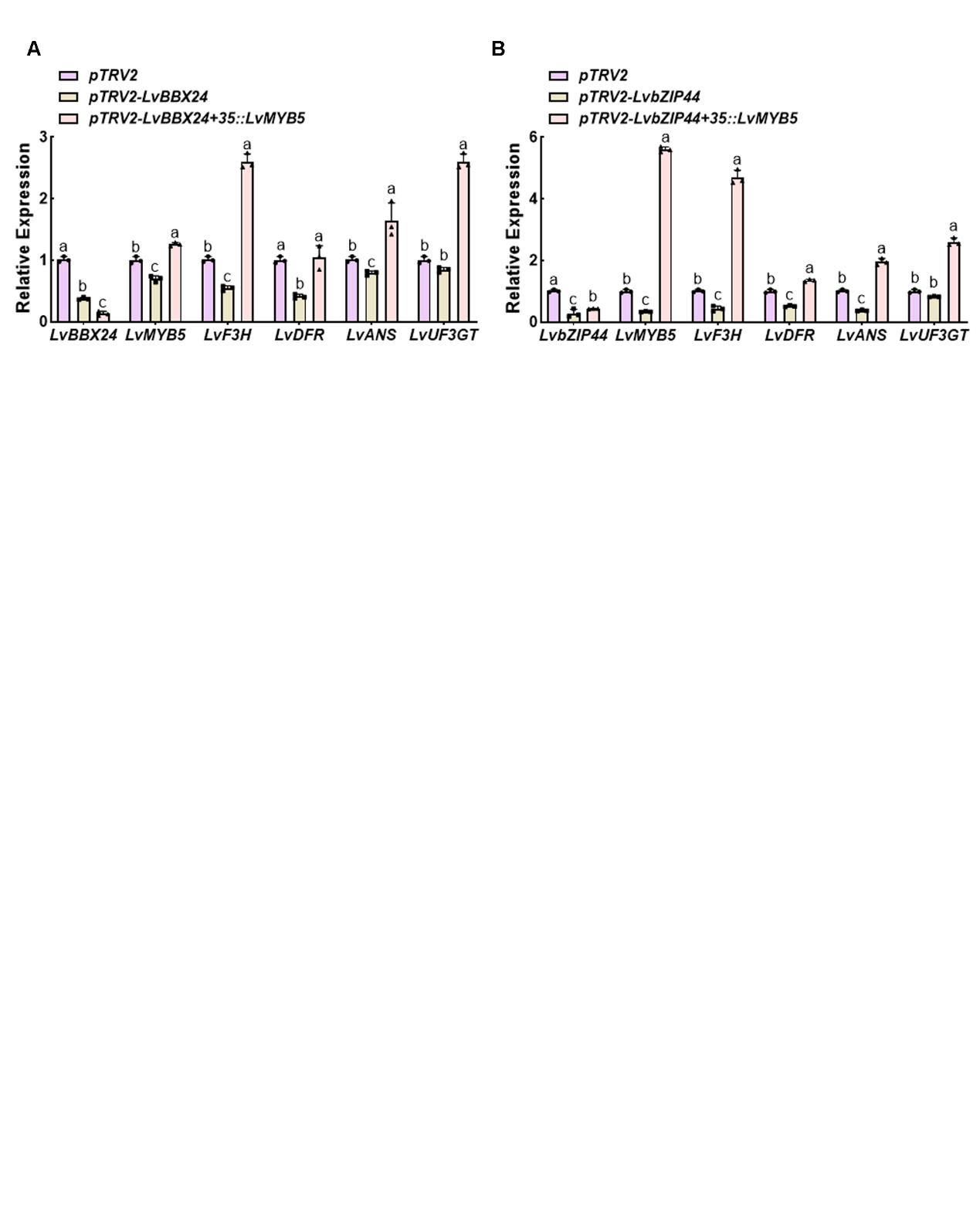


**Figure S5 Silencing LvBBX24 and LvbZIP44 plants to complement the LvMYB5 gene. A** On the basis of silencing LvBBX24, overexpressing the potential downstream gene *LvMYB5*. **B** On the basis of silencing LvbZIP44, overexpressing the potential downstream gene *LvMYB5*. Data are the means of three biological replicates ± SD. Different letters indicate significant differences by Tukey s-b (K) test with P < 0.05.


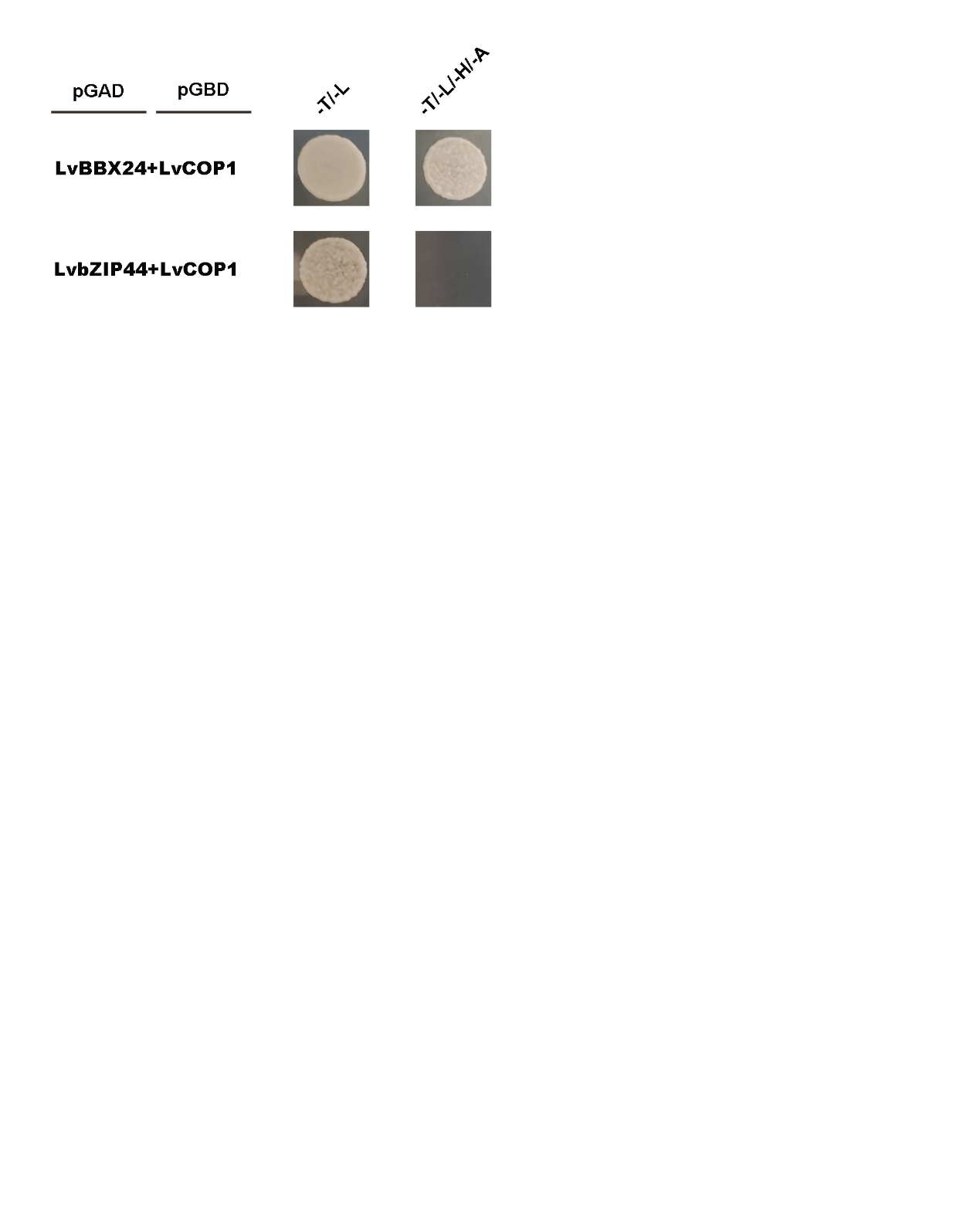


**Figure S6 Y2H validates the interaction of LvBBX24-LvCOP1, LvbZIP44-LvCOP1** Y2H experiment involved fusion of LvBBX24 and LvbZIP44 genes into the pGAD vector, while LvCOP1 was fused into the pGBD vector. Yeast cells were cultured on SD medium lacking Trp and Leu (-T/-L) for selection, and on SD medium lacking Trp, Leu, His, and Ade (-T/-L/-H/-A) for further confirmation.

**
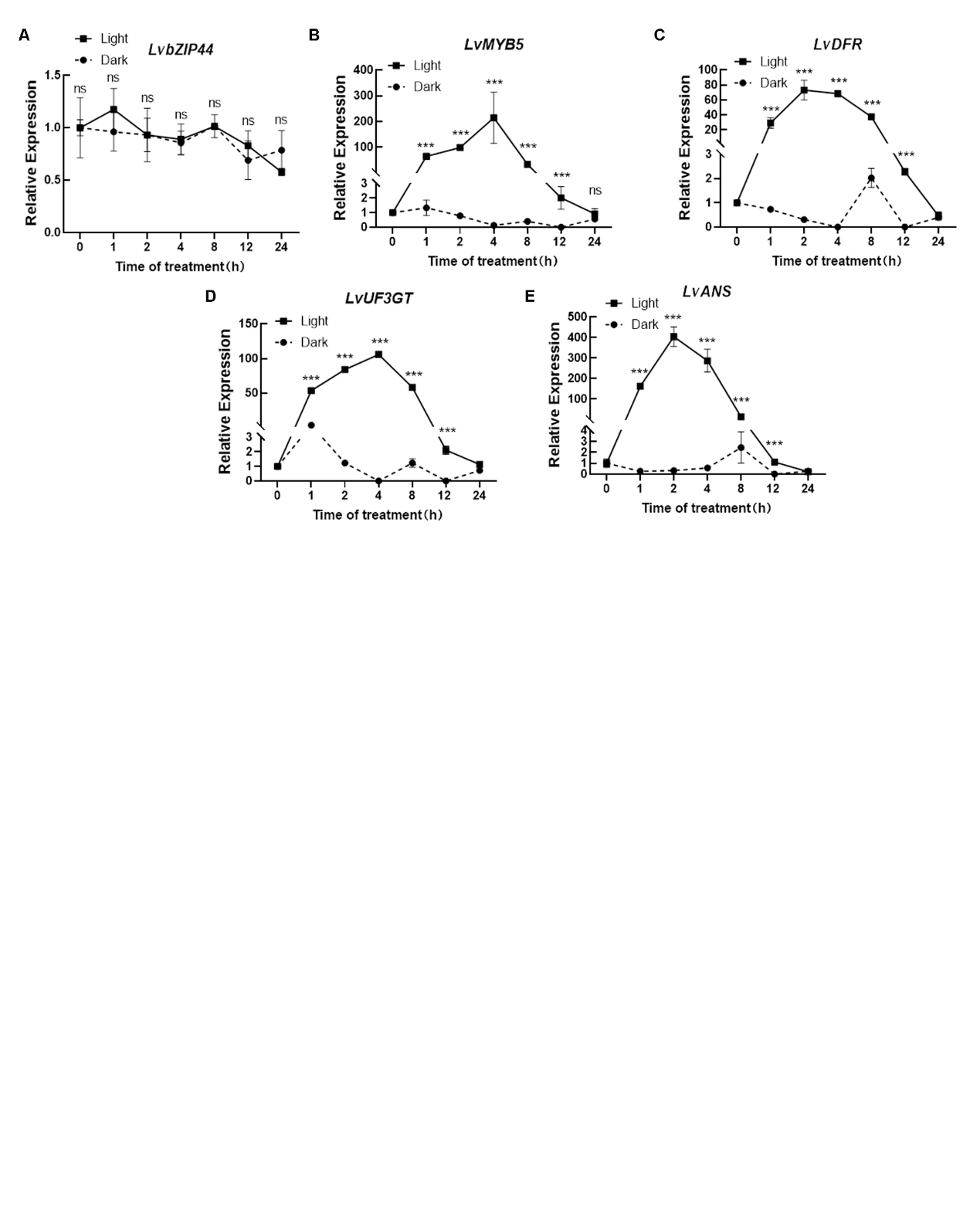
**

**Figure S7 Relative expression patterns of *LvbZIP44* and anthocyanin-related genes in ‘Viviana’ lily petals during light treatment.**

**
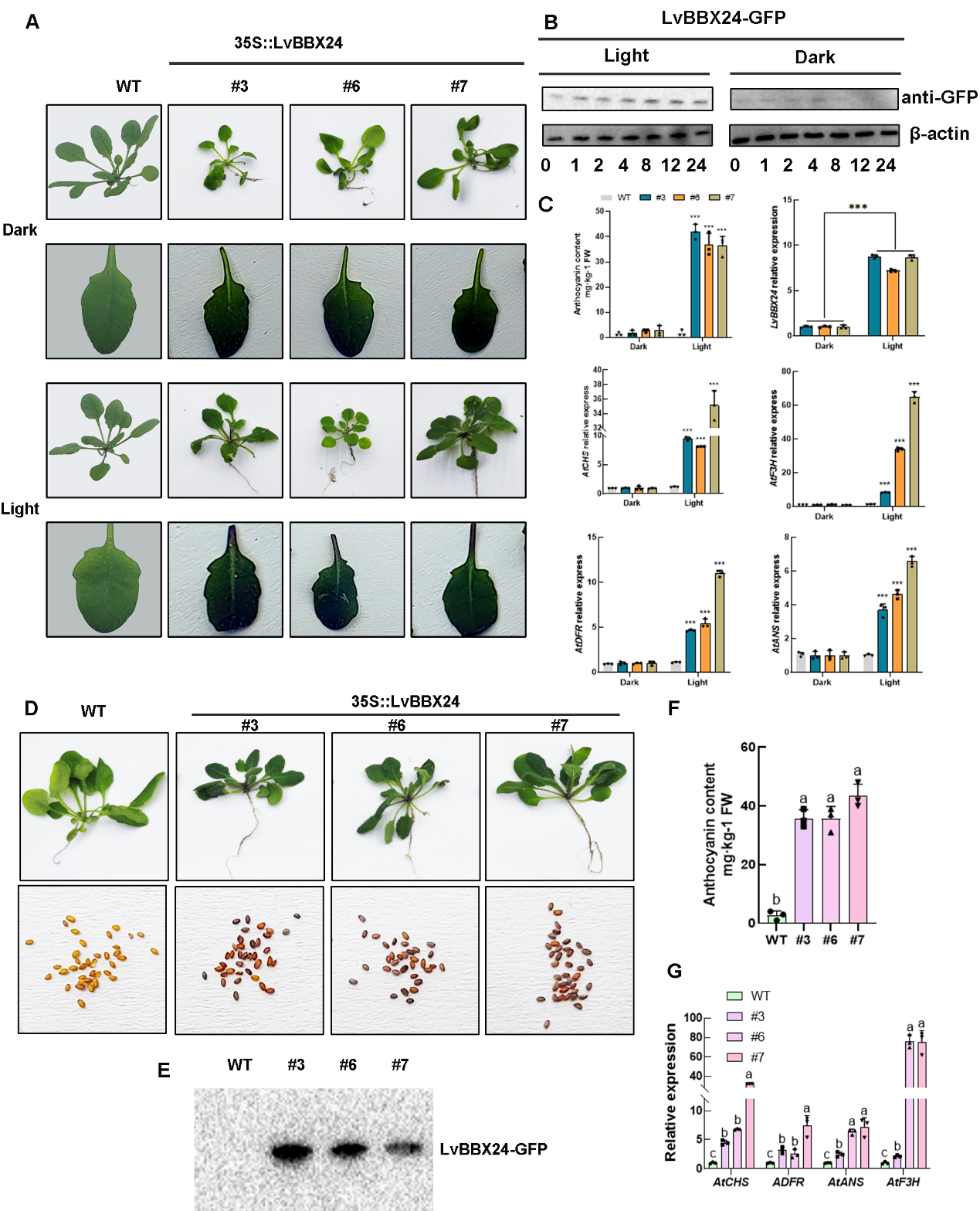
**

**Figure S8 LvBBX24 heterologous expression of Arabidopsis phenotypes and light treatment data. A** Heterologous overexpression LvBBX24 gene in Arabidopsis thaliana that was protected from light for 5 days and exposed to light for 20 days. **B** LvBBX24 heterologous overexpression in Arabidopsis thaliana and changes in LvBBX24-GFP protein abundance after one hour of dark treatment. **C** Anthocyanin content and expression levels of anthocyanin-related genes. **D** Phenotypes of LvBBX24 heterologous overexpression in Arabidopsis thaliana. **E** LvBBX24-GFP was detected in overexpressed Arabidopsis thaliana. **F** Anthocyanin content of heterologously expressed Arabidopsis thaliana. **G** Expression levels of anthocyanin-related genes.


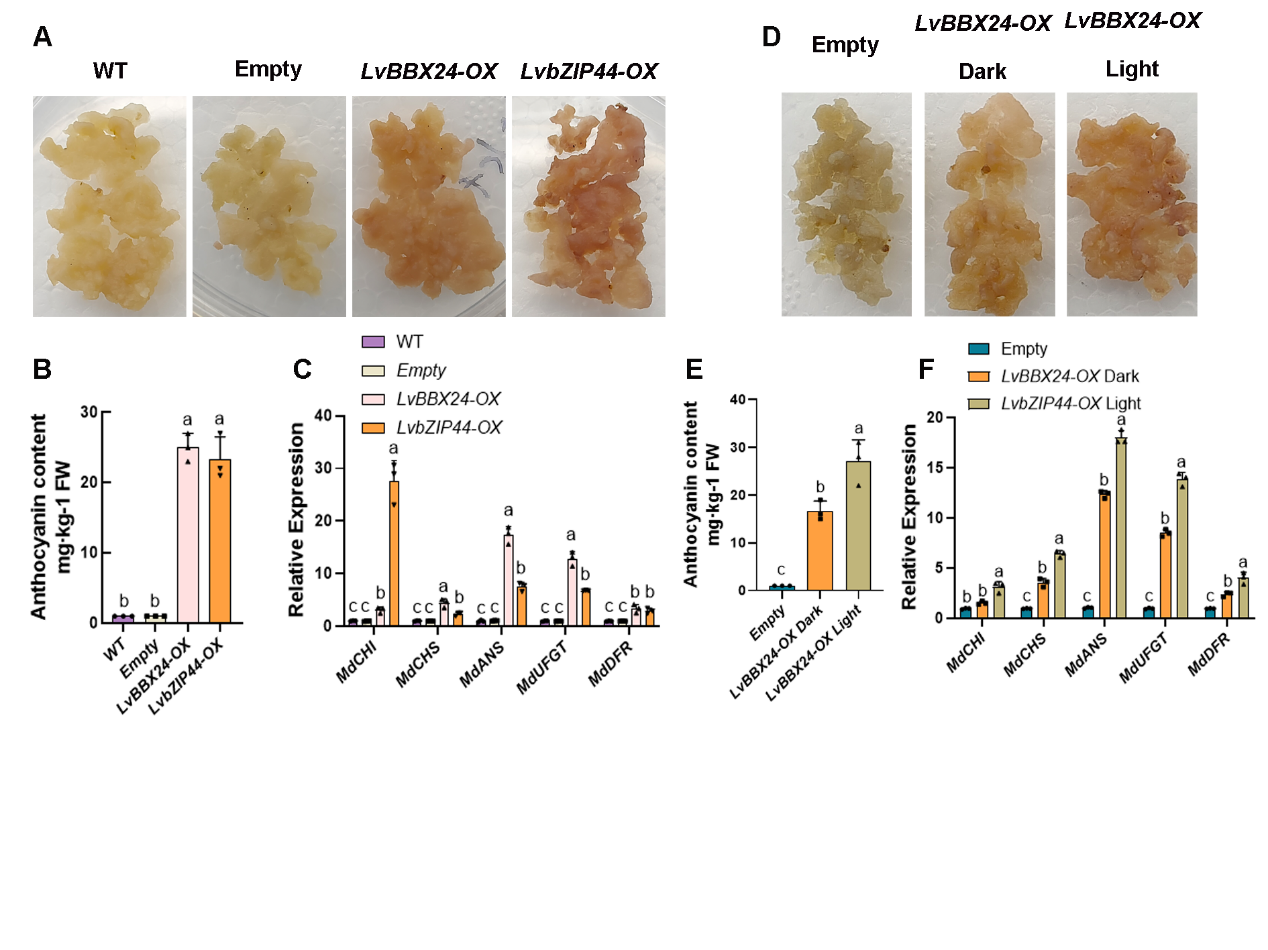


**Figure S9 LvBBX24 and LvbZIP44 heterologous expression of apple calli and heterologous expression of LvBBX24 in apple callus treated with light. A** Transformation of 35S::Empty, 35S::*LvBBX24* and 35S::*LvbZIP44* into apple callus through Agrobacterium infection. **B** Anthocyanin content in wild-type apple, 35S::Empty, 35S::*LvBBX24*, 35S::*LvbZIP44* overexpressed apple callus. **C** Expression levels of anthocyanin biosynthetic structural genes in wild-type apple, 35S::Empty, 35S::LvBBX24, 35S::LvbZIP44 overexpressed apple callus tissue. **D** Light and dark treatment of apple callus of 35S::*LvBBX24*. **E** Anthocyanin content of apple callus treated with light and dark 35S::*LvBBX24*. **F** Expression levels of anthocyanin biosynthesis structural genes in apple callus treated with light and dark 35S::LvBBX24.


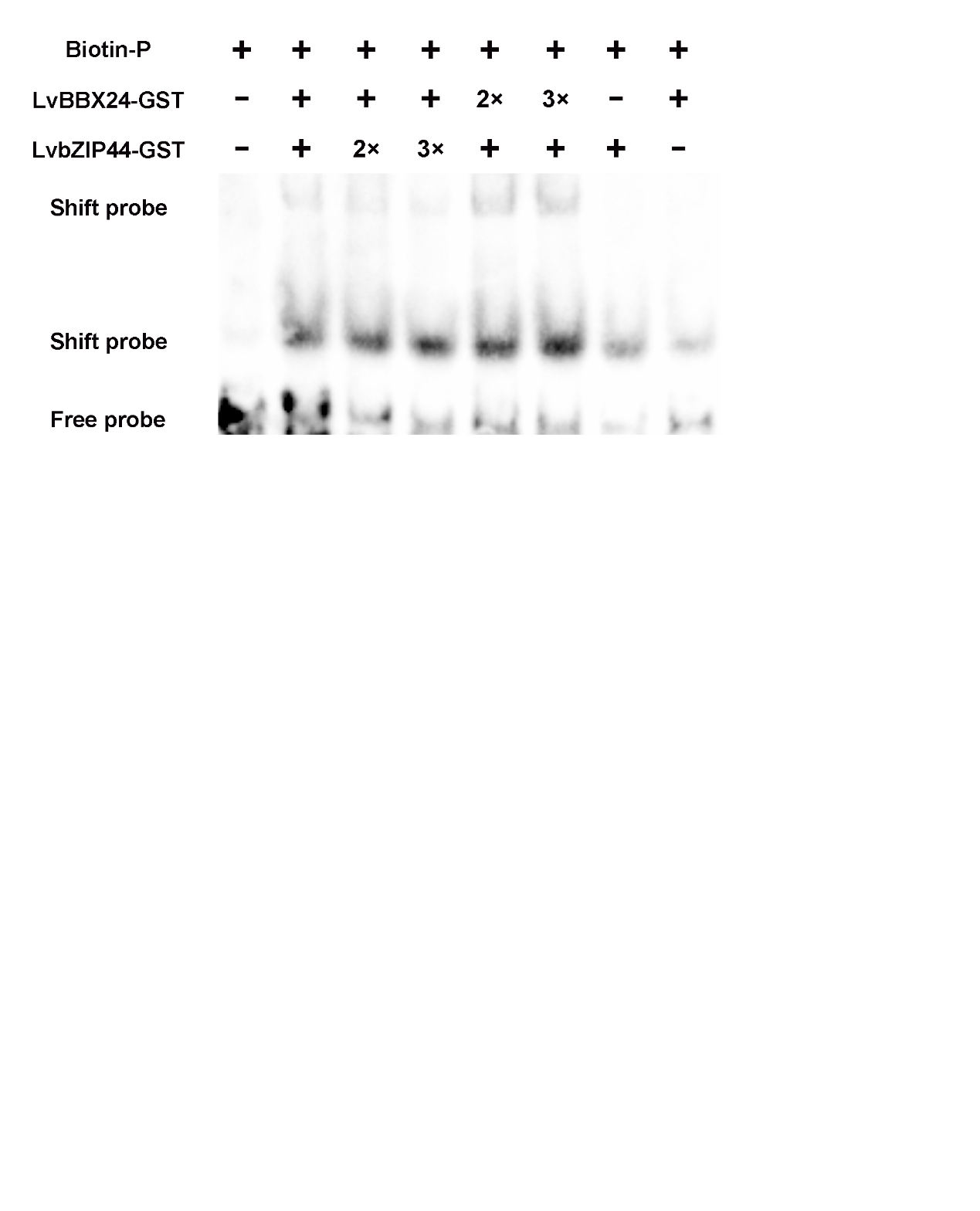


**Figure S10** EMSA shows that there are two shift bands in the LvBBX24-LvbZIP44 complex binding probe. LvBBX24+LvbZIP44 complex with G-BOX.
