# Supplementary figures and images for: Transcription factors LvBBX24 and LvbZIP44 coordinated anthocyanin accumulation in response to light in lily petals
Source: Hortic Res. 2024 Jul 30;11(10):uhae211. doi: 10.1093/hr/uhae211 (PMC11450212; doi:10.1093/hr/uhae211)

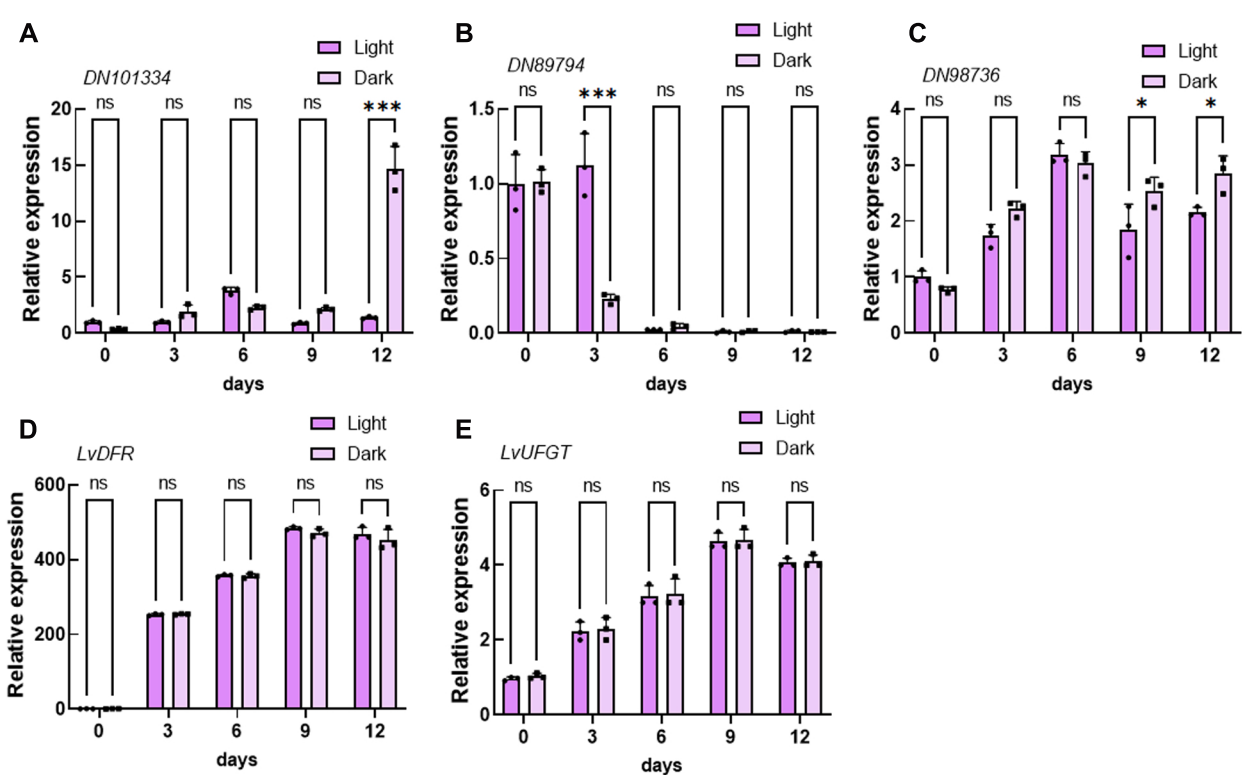

**F**

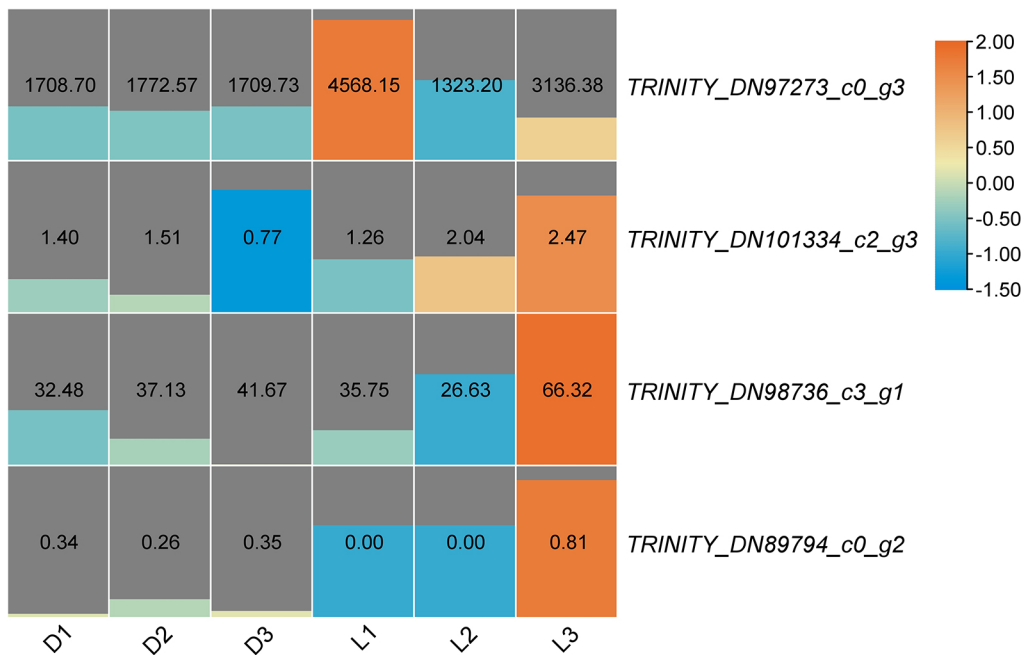

Supplement: Web_Material_uhae211 [file web_material_uhae211.zip › Supporting Figure S1.pdf]

**A**

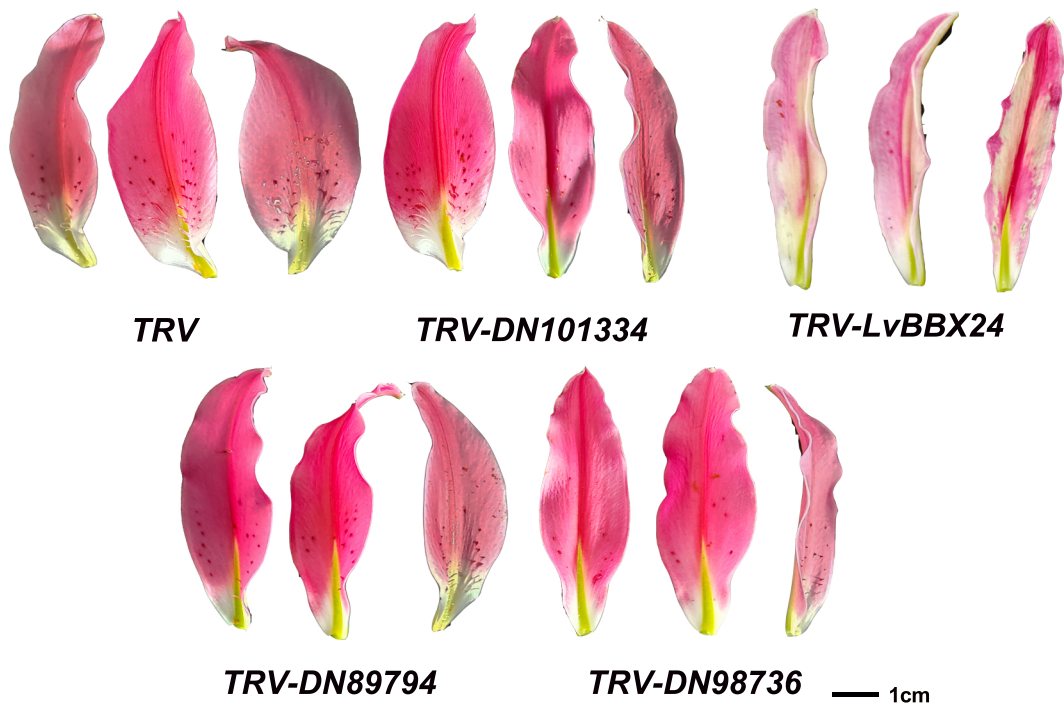

**B**

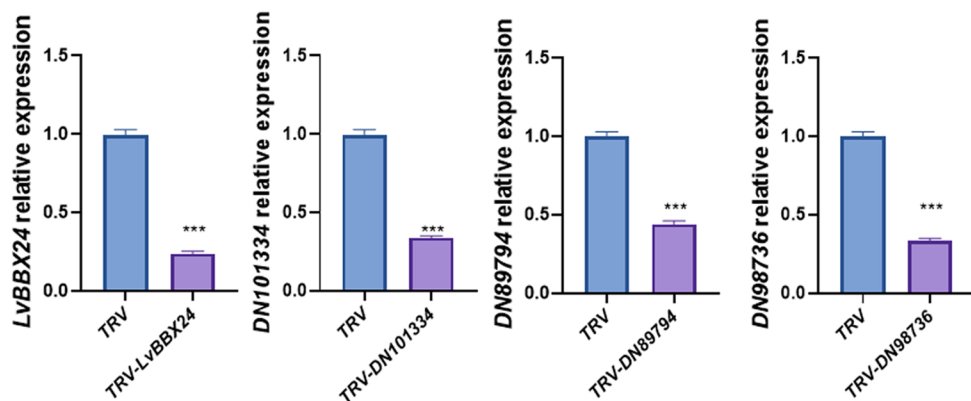

**C**

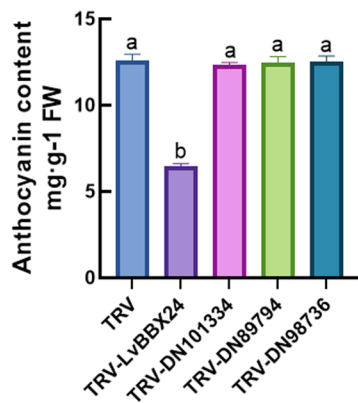

**D**

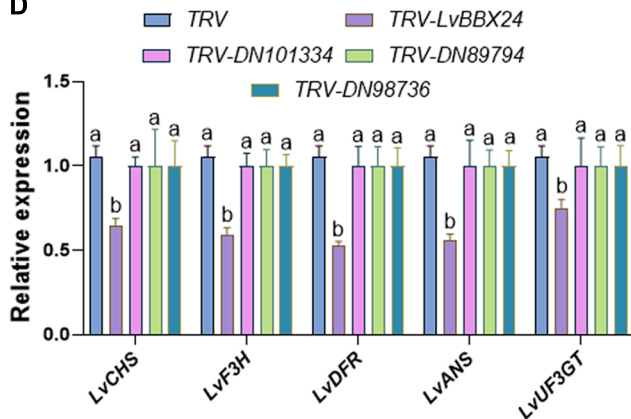

Supplement: Web_Material_uhae211 [file web_material_uhae211.zip › Supporting Figure S2.pdf]

A

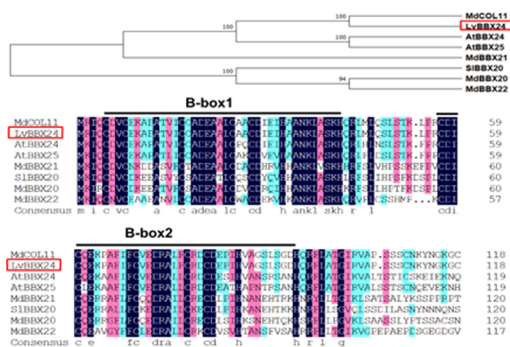

B

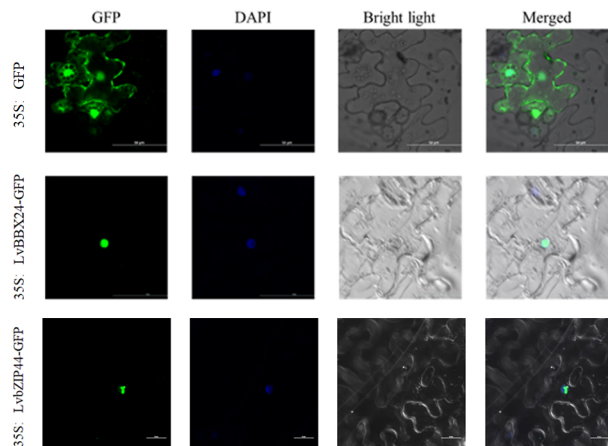

C

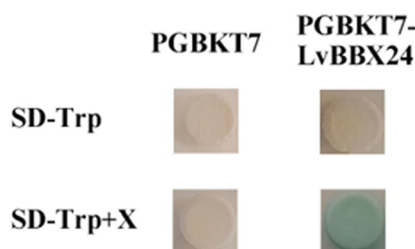

D

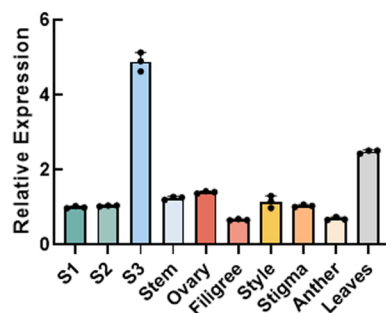

E

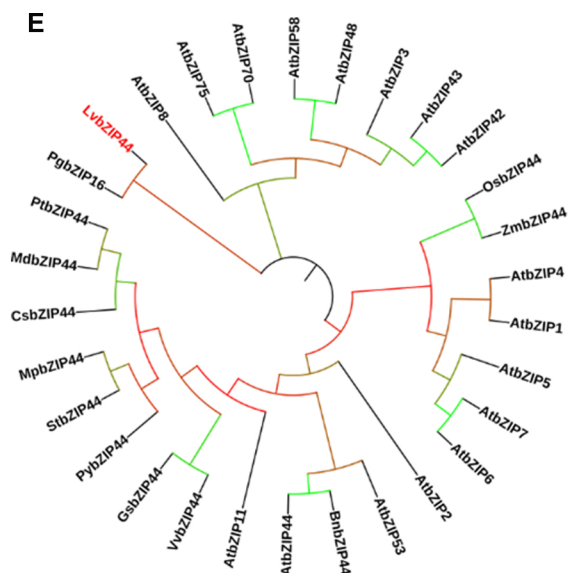

F

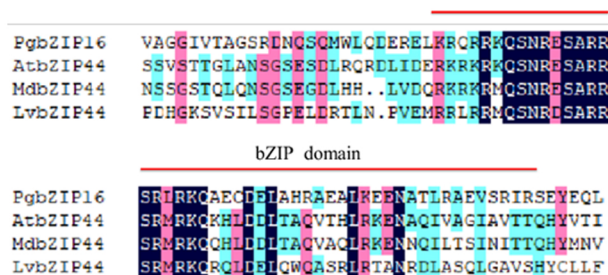

Supplement: Web_Material_uhae211 [file web_material_uhae211.zip › Supporting Figure S3.pdf]

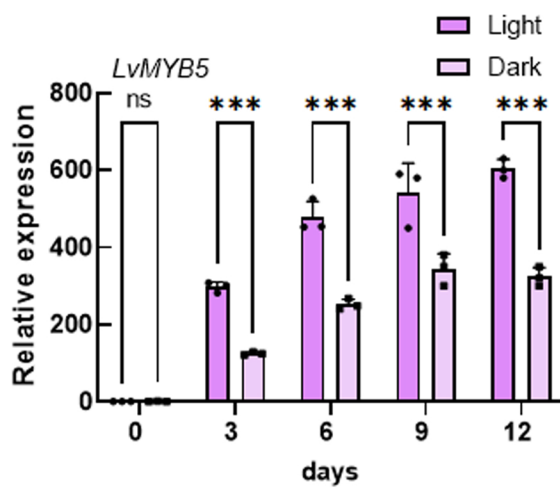

Supplement: Web_Material_uhae211 [file web_material_uhae211.zip › Supporting Figure S4.pdf]

A

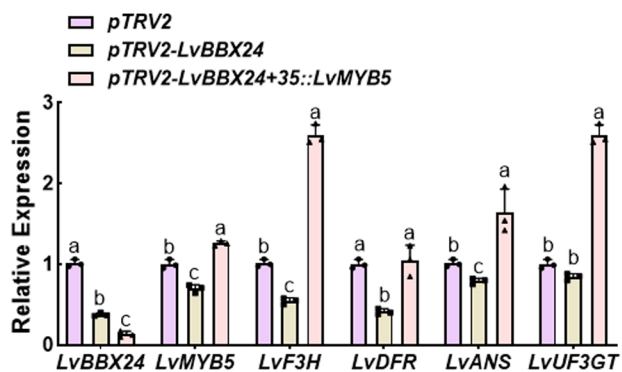

B

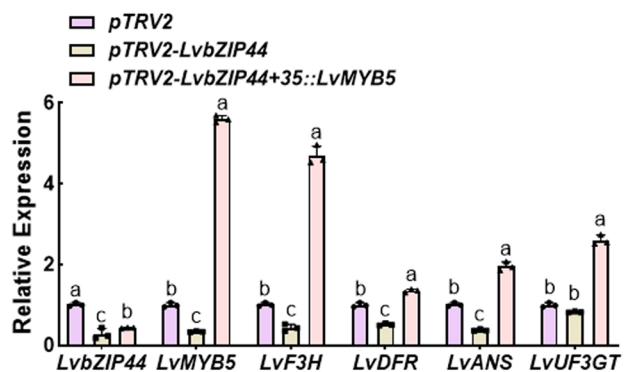

Supplement: Web_Material_uhae211 [file web_material_uhae211.zip › Supporting Figure S5.pdf]

| pGAD            | pGBD | -Ti-L                                                                             | -Ti-L-H/A                                                                         |
|-----------------|------|-----------------------------------------------------------------------------------|-----------------------------------------------------------------------------------|
| LvBBX24+LvCOP1  |      | 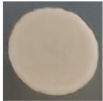 | 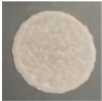 |
| LvbZIP44+LvCOP1 |      | 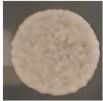 | 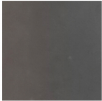 |

Supplement: Web_Material_uhae211 [file web_material_uhae211.zip › Supporting Figure S6.pdf]

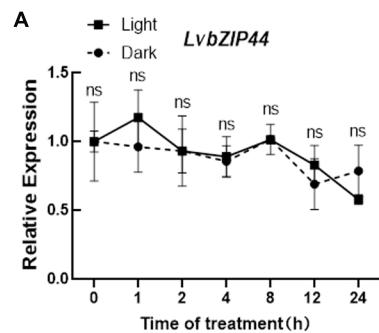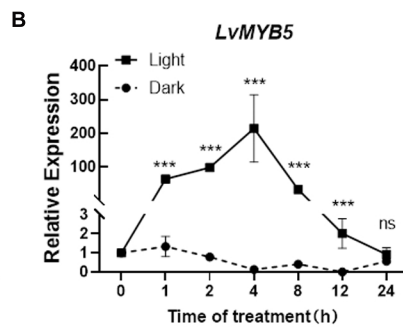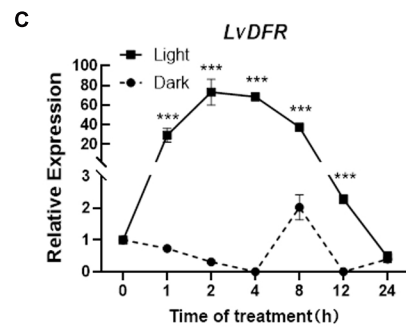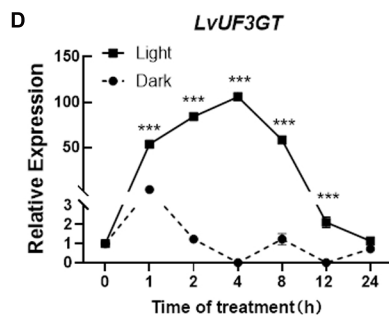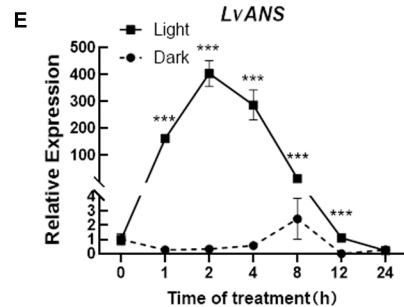

Supplement: Web_Material_uhae211 [file web_material_uhae211.zip › Supporting Figure S7.pdf]

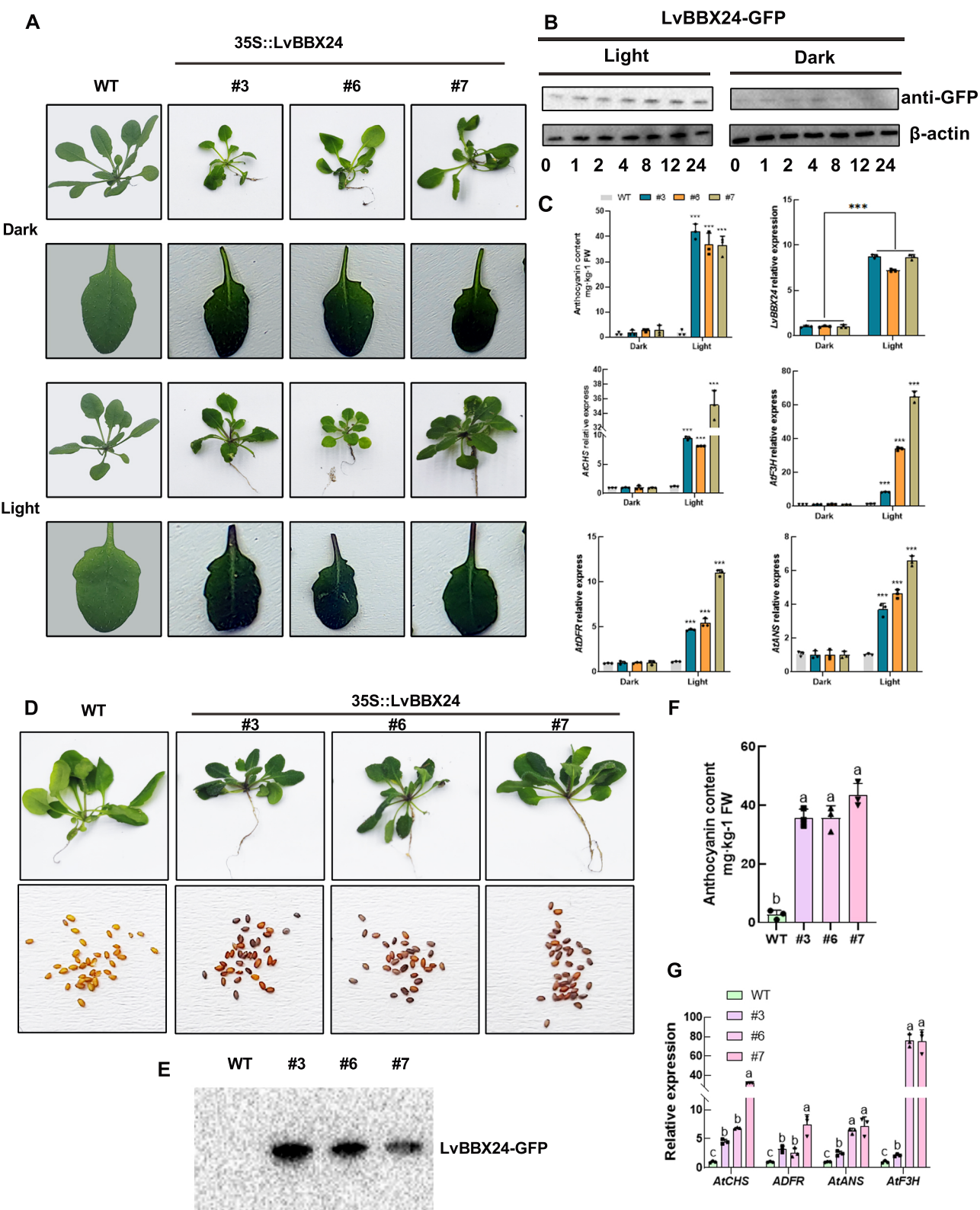

Supplement: Web_Material_uhae211 [file web_material_uhae211.zip › Supporting Figure S8.pdf]

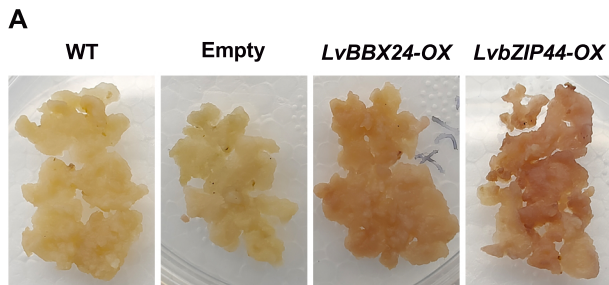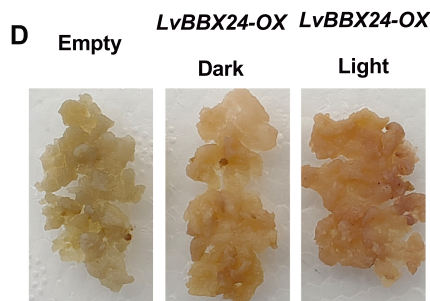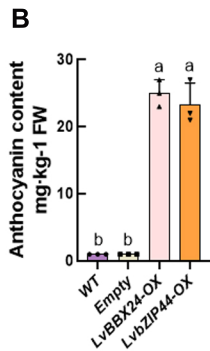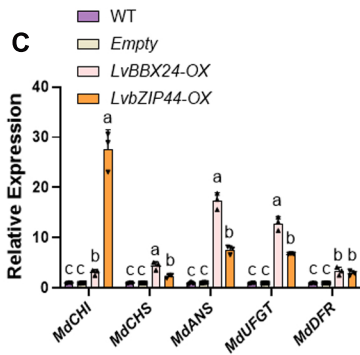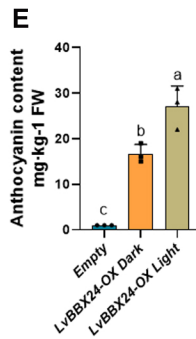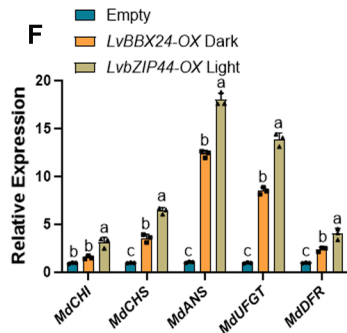

Supplement: Web_Material_uhae211 [file web_material_uhae211.zip › Supporting Figure S9.pdf]

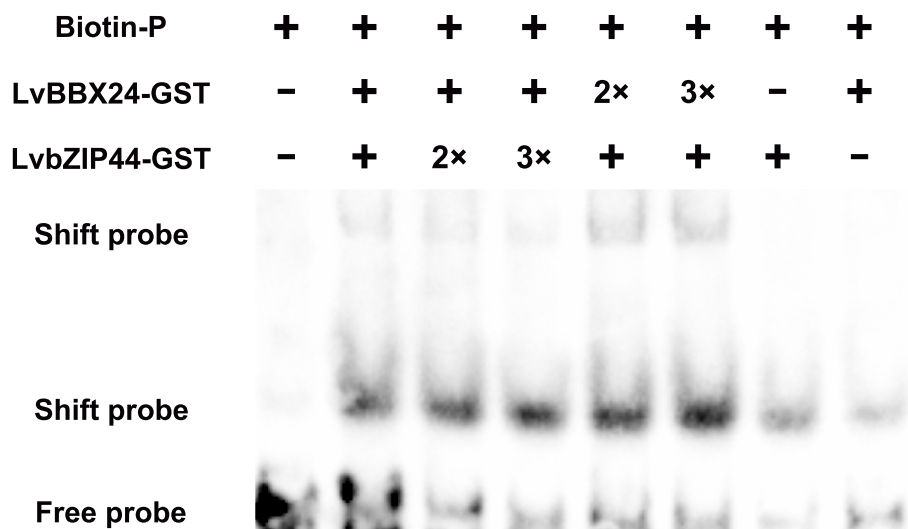

Supplement: Web_Material_uhae211 [file web_material_uhae211.zip › Supporting Figure S10.pdf]
